# Synchronous force and Ca^2+^ measurements for repeated characterization of excitation-contraction coupling in human myocardium

**DOI:** 10.1038/s42003-024-05886-3

**Published:** 2024-02-22

**Authors:** Zhengwu Sun, Kun Lu, Christine Kamla, Petra Kameritsch, Thomas Seidel, Andreas Dendorfer

**Affiliations:** 1 Walter-Brendel-Centre of Experimental Medicine, University Hospital, Ludwig-Maximilians-University Munich, Munich, Germany; 2 Department of Cardiac Surgery, University Hospital, Ludwig-Maximilians-University Munich, Munich, Germany; 3 DZHK (German Center for Cardiovascular Research), Partner site Munich Heart Alliance, Munich, Germany; 4 Institute of Cellular and Molecular Physiology, Friedrich-Alexander University Erlangen-Nürnberg, Erlangen, Germany

**Keywords:** Experimental models of disease, Fluorescent dyes

## Abstract

Dysfunctional Ca^2+^ signaling affects the myocardial systole and diastole, may trigger arrhythmia and cause transcriptomic and proteomic modifications in heart failure. Thus, synchronous real-time measurement of Ca^2+^ and force is essential to investigate the relationship between contractility and Ca^2+^ signaling and the alteration of excitation-contraction coupling (ECC) in human failing myocardium. Here, we present a method for synchronized acquisition of intracellular Ca^2+^ and contraction force in long-term cultivated slices of human failing myocardium. Synchronous time series of contraction force and intracellular Ca^2+^ were used to calculate force-calcium loops and to analyze the dynamic alterations of ECC in response to various pacing frequencies, post-pause potentiation, high mechanical preload and pharmacological interventions in human failing myocardium. We provide an approach to simultaneously and repeatedly investigate alterations of contractility and Ca^2+^ signals in long-term cultured myocardium, which will allow detecting the effects of electrophysiological or pharmacological interventions on human myocardial ECC.

## All

The authors present a Ca^2+^ imaging method based on visible light and ratiometric dye, which permits motion-corrected assessment of Ca^2+^ transients in parallel with contraction force in cultured slices of human living myocardium.

## Introduction

Dysfunction of intracellular Ca^2+^ handling substantially affects myocardial systole and diastole, and even causes transcriptomic and proteomic alterations in ventricular myocardium from patients with heart failure^1,2^. Moreover, reduced Ca^2+^ uptake by sarcoplasmic reticulum Ca^2+^-ATPase (SERCA) and increased Ca^2+^ leakage by ryanodine receptor, and increased sodium-calcium exchanger (NCX) cause the abnormality of excitation-contraction coupling (ECC) in heart failure^2–5^. Thus, the real-time synchronized measurement of Ca^2+^ and force is critical to investigate pathological ECC in human failing myocardium.

Previous measurements of myocardial force and Ca^2+^ were mainly based on acutely isolated animal myocardium at room temperature^6–8^. The evaluation of Ca^2+^ and force frequently fails to predict the alteration of ECC in the authentic human tissue due to the instability of short-term animal myocardial models. For human myocardium, techniques of myocardial tissue culture have recently been established with the development of biomimetic culture conditions that support the viability and continuous beating of thin myocardial tissue slices over months^9,10^, thus enabling multiple consecutive force and Ca^2+^ measurements^11^.

Intracellular Ca^2+^ is commonly assessed by loading cardiomyocytes with fluorescent Ca^2+^ indicators and subsequent time-series imaging on a microscope during which the isolated cells or tissues are stimulated by application of an electrical field. Ca^2+^ indicators can be divided into two classes: (1) single-excitation/single-emission indicators, which change their overall fluorescent intensity when binding Ca^2+^, and (2) ratiometric indicators, which change their excitation or emission spectrum when binding Ca^2+^. Compared to single-excitation wavelength Ca^2+^ indicators, such as Fluo-4 or Fluo-8^12,13^, ratiometric fluorescence measurements may correct for inconsistent dye loading, bleaching, thickness of cells or tissues, and motion artifacts. However, the best-established ratiometric Ca^2+^ indicators (Fura-2 and Indo-1) have limitations, including lower sensitivity, alternating UV excitation, and incompatibility with high-throughput screening filter sets^14,15^. To avoid these disadvantages, CalRed R525/650 AM (CalRed), a visible light excitable (488 nm) ratiometric Ca^2+^ indicator with an emission signal increasing at 525 nm and decreasing at 650 nm upon Ca^2+^ binding was considered here for synchronized force and Ca^2+^ measurements^16^.

In this study, we investigated human myocardial slices after 3–4 weeks of cultivation in biomimetic culture chambers (BMCCs). Because myocardial tissue preparations are very sensitive to mechanical manipulation, temperature and pH changes, we were able to utilize the force sensor and length adjustment integrated in BMCCs to investigate effects on the Ca^2+^ signal. We aimed for a method that would permit synchronized and repeated force and Ca^2+^ measurement within BMCCs. Finally, the complete protocol, including cultivation, dye loading, functional assessments with programmed stimulation and drug application was performed without mechanical manipulation of the tissues and with the possibility to continue cultivation for long-term treatments.

## Results

### Analysis of synchronized force-calcium kinetics and relationship

**Fig. 1:**
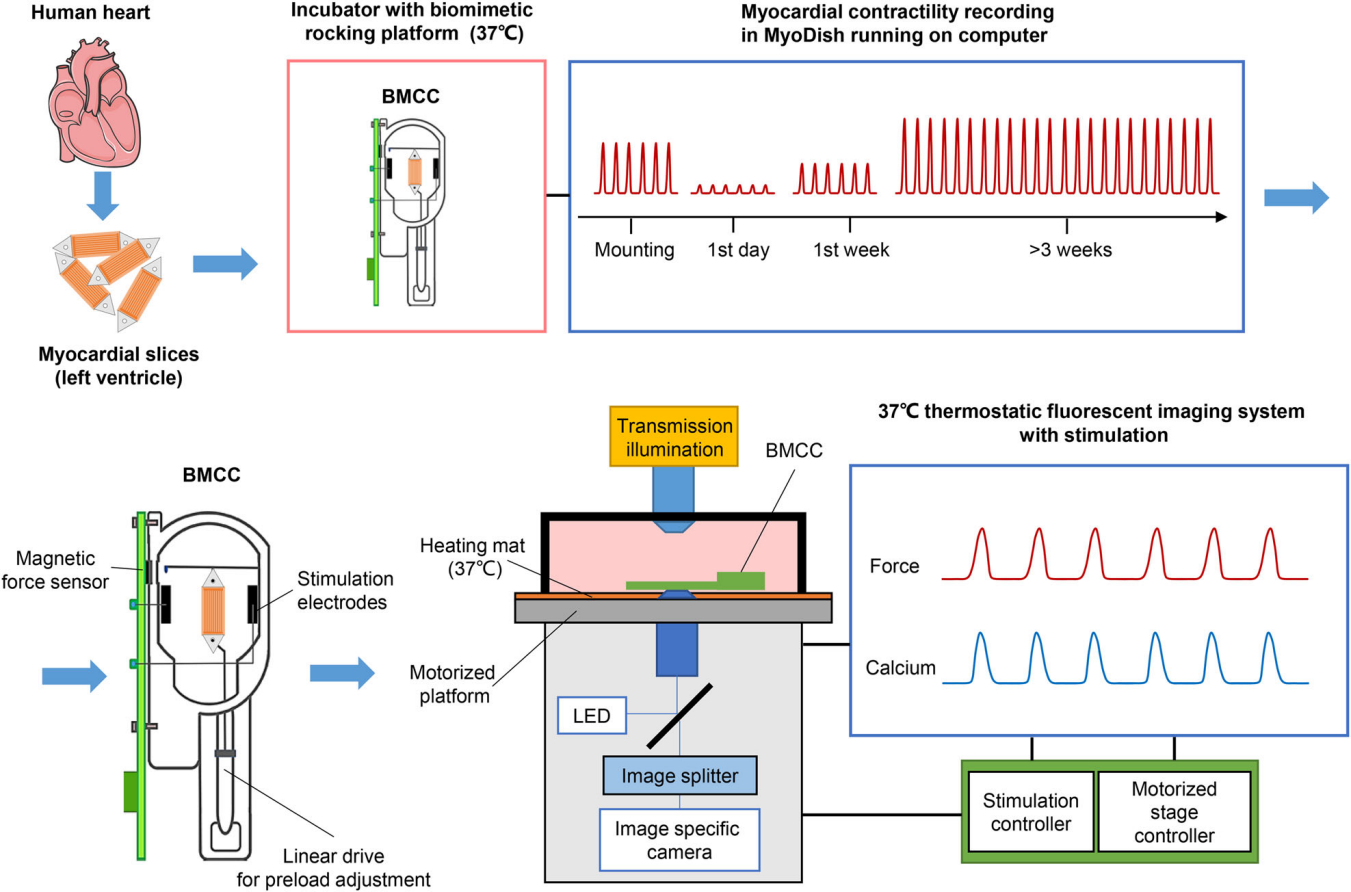
Schematic illustration of human myocardial slice acquisition and long-term cultivation and synchronized force and calcium measurements in biomimetic culture chambers (BMCCs). Left ventricular tissue slices were acquired and cultured in vitro for >3 weeks. BMCCs were transferred to a custom-made fluorescence microscope system for synchronized measurement of contractility and calcium signal under electrical stimulation. The heart illustration is taken from Servier Medical Art, provided by Servier and licensed under a Creative Commons Attribution 3.0 Unported License.

**Fig. 2:**
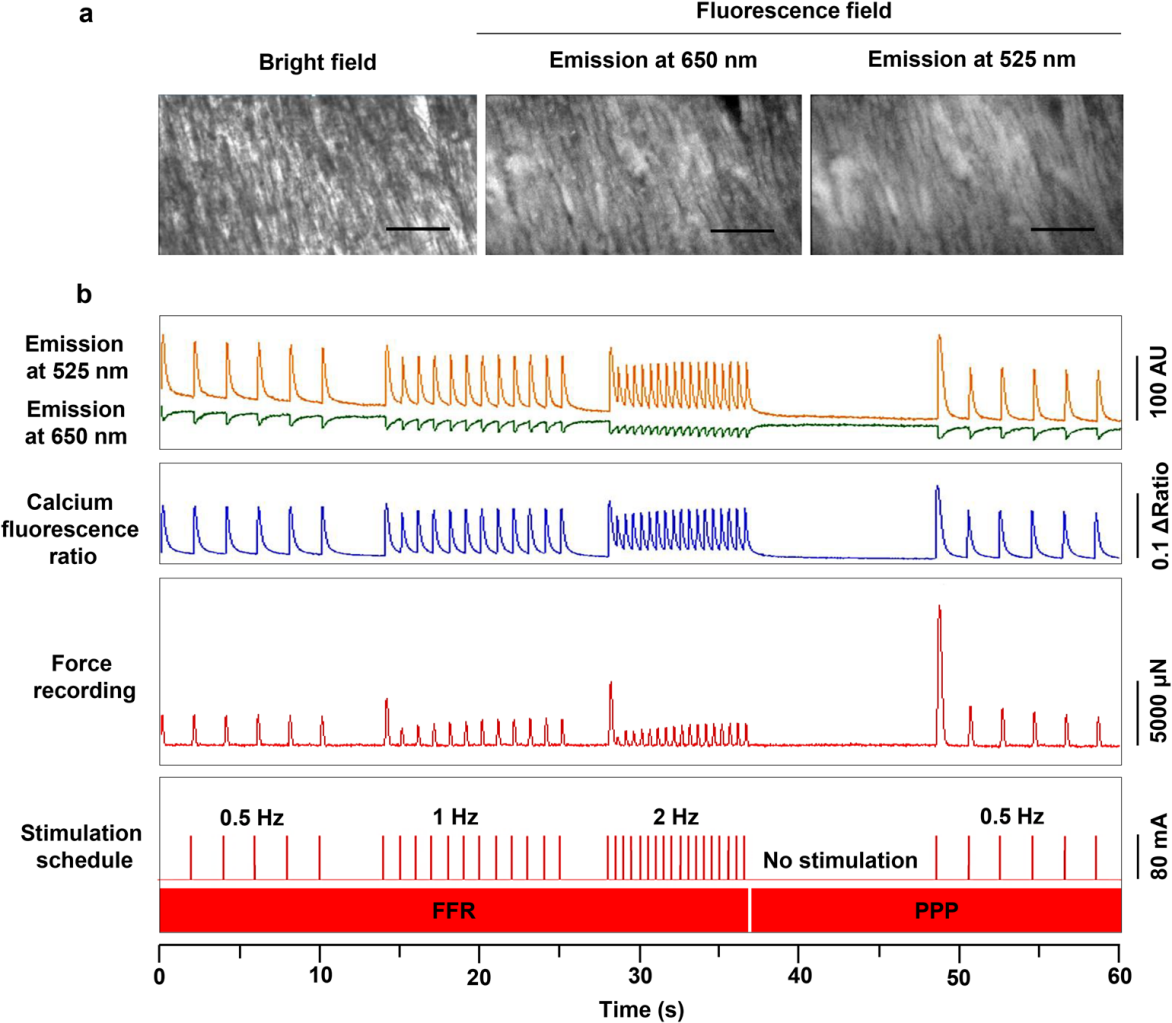
Simultaneous measurements of force and calcium in human myocardium. **a** Microscopic images of beating myocardium after CalRed loading in the bright and fluorescent fields (20× magnification, scale bar 135 μm). **b** Calcium transients and ratio from CalRed (high Ca_i_^2+^ at 525 nm, low Ca_i_^2+^ at 650 nm), as well as force transients in the customized stimulation schedule for force-frequency relation (FFR) and post-pause potentiation (PPP) evaluation. AU represents arbitrary units.

**Fig. 3:**
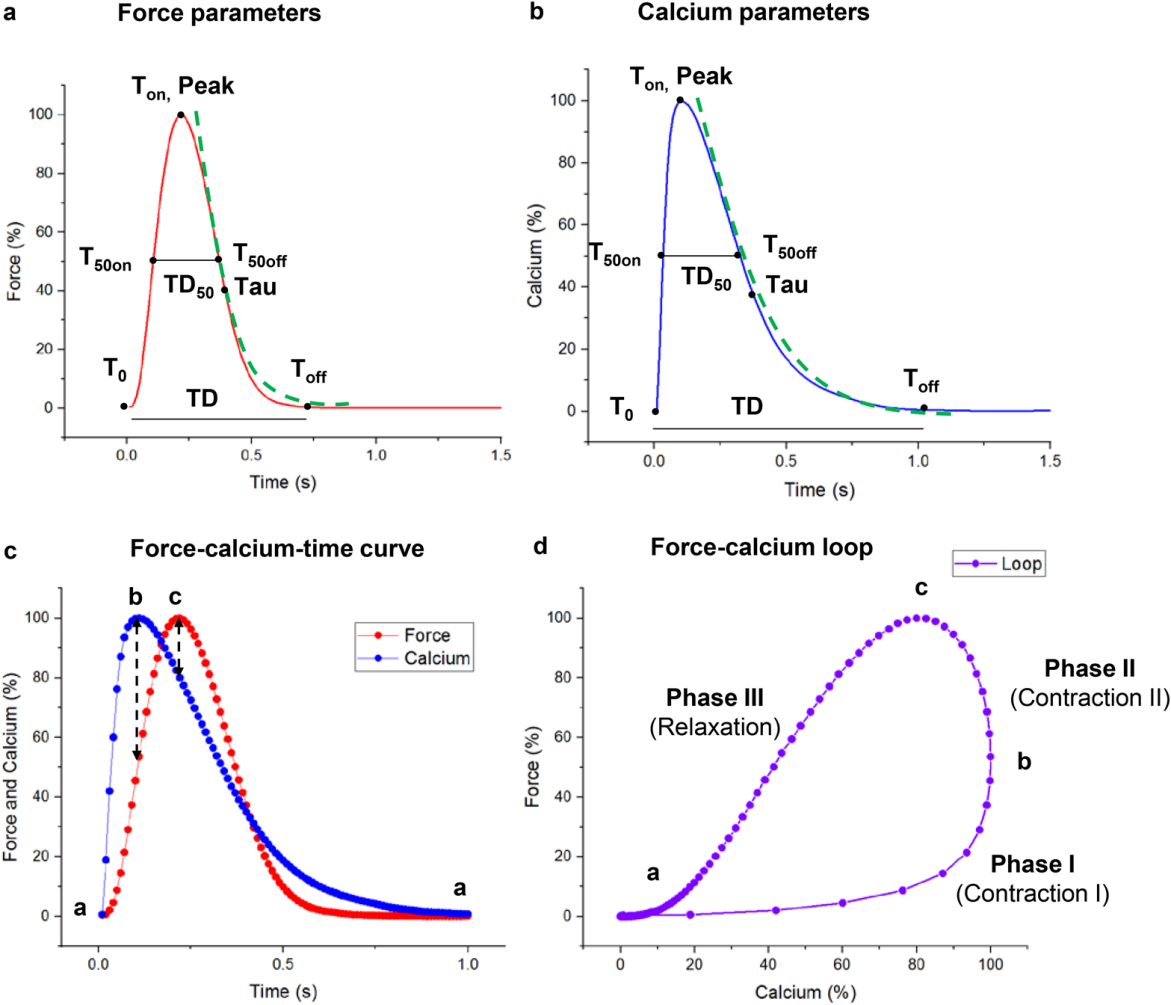
Synchronized analysis of force and calcium kinetics. **a**, **b** Schematic force and calcium kinetics (peak amplitude, tau, T_0_, T_50on_, T_on_, T_50off_, T_off_, TD_50_, and TD), dash green lines represent single exponential fitted decay curve for tau. **c** Representative force-time and calcium-time curves (red, blue, point a = diastolic baseline, point b = maximum calcium point in systole, point c = maximum force point in systole), the time interval of dots is 10 ms. **d** Representative force-calcium loop (“a–b” = contraction phase I; “b–c” = contraction phase II; “c–a” = relaxation phase), the density of the dots is inversely related to the speed of transients.

Myocardial slices from 10 patients were used for this study, patient characteristics are listed in Supplementary Table 1. Forces developed by the tissues are given in mN, but due to the uniform cross section of the slices (width 7 mm × thickness 0.3 mm), the mechanical stress is linearly correlated to the developed force (1 mN corresponds to 0.48 mN/mm^2^). Corresponding changes of force and intracellular Ca^2+^ (Ca_i_^2+^) were acquired simultaneously and in direct relationship to the electrical stimulation of the tissue. To assess the importance of pre-beat intervals, stimulation was applied at different pacing frequencies (0.5 Hz for 10 s, 1 Hz for 12 s, and 2 Hz for 10 s). Subsequently, stimulation was stopped for 12 s and resumed at 0.5 Hz for determination of post-pause potentiation (PPP). Measurements of diastolic force and Ca_i_^2+^ were taken during the extended pause interval in a custom-built microscopy system (Fig. 1 and 2). Force and Ca_i_^2+^ kinetics, including peak amplitude, tau (*τ*, decay constant), T_50on_ (time to half peak), T_on_ (time to peak), T_50off_ (time of half decay), T_off_ (time of decay), TD_50_ (duration at half peak), and TD (transient duration), were analyzed for the terminal contraction at 0.5-2 Hz, and for the initial peak after an extended pause (Fig. 3a, b). The rise of Ca_i_^2+^ started within the 10 ms period of the stimulation impulse, whereas there was an average 18.7 ± 1.3 ms delay in myocardial contraction (point a = baseline in diastole). The peak of Ca_i_^2+^ occurred 112 ± 5.9 ms earlier than the maximum force development when myocardium was paced at 0.5 Hz. A detectable increase in Ca_i_^2+^ persisted longer than the actual developed systolic force. The dynamic relationship between contractility and Ca_i_^2+^ is visualized in the force-calcium loop (Fig. 3c, d).

### Time- and preload-dependent alterations of force and Ca_i_^2+^

**Fig. 4:**
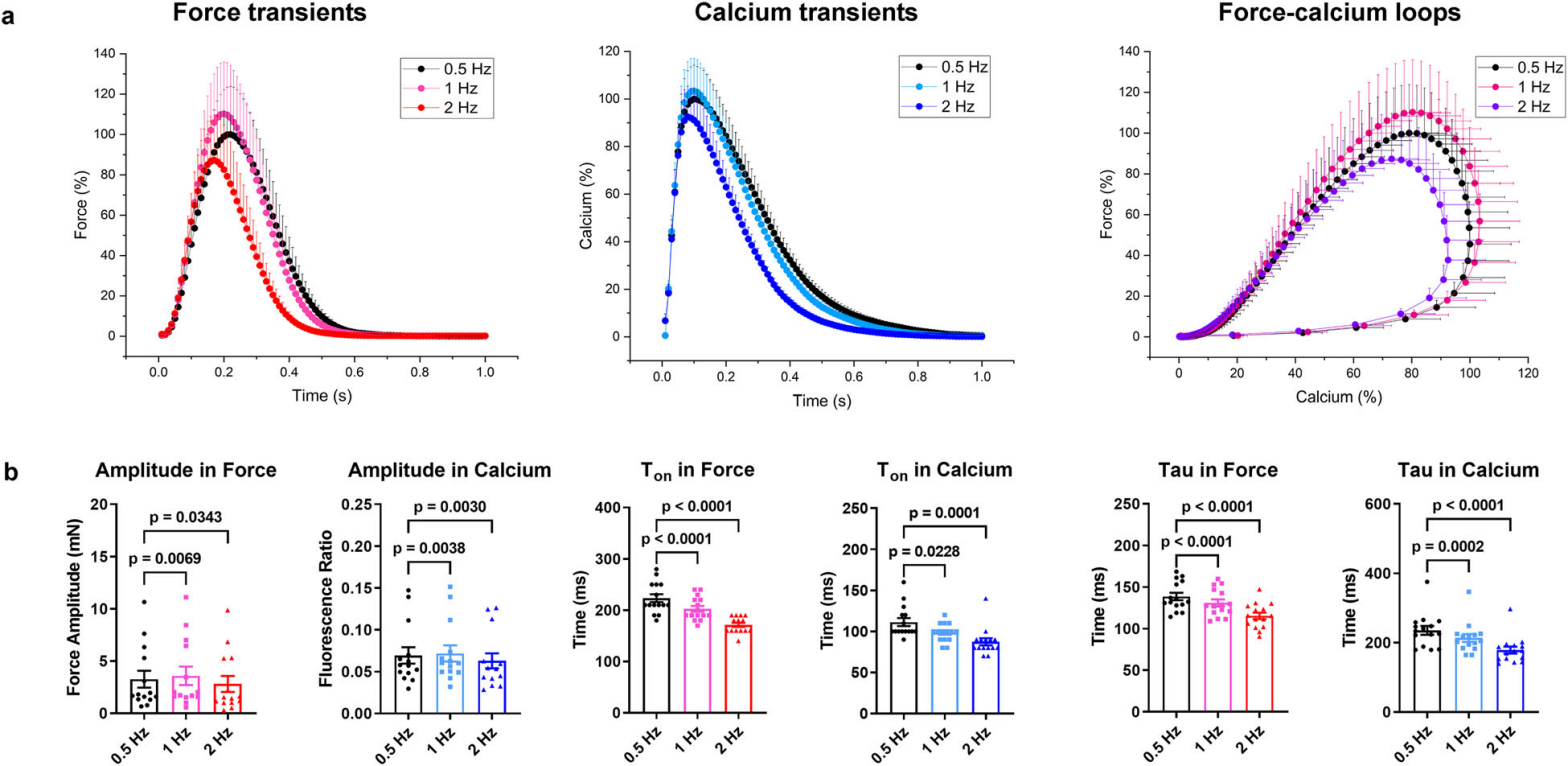
Alterations of force and calcium transients at different pacing frequencies. **a** Normalized average contraction force and calcium and force-calcium loops in human myocardial slices (0.5 Hz, 1 Hz, 2 Hz; *n* = 5 patients, 14 slices). **b** Amplitude (difference between peak and baseline values), T_on_, and tau in force and calcium transients at increasing pacing frequencies (amplitude, *n* = 5 patients, 14 slices; T_on_ and tau, *n* = 5 patients, 15 slices). Data are presented as mean and SEM. Statistical analysis is performed by one-way ANOVA with Dunnett’s multiple comparisons posttest versus 0.5 Hz in force-frequency and calcium-frequency relationships. A *p* < 0.05 was defined as the significance cutoff.

Loss of contractility at high heart rates, and general negative force-frequency commonly occur in human failing myocardium. Since the human heart slices had partially recovered during 3–4 weeks of culture prior to the experiment, the peak amplitudes of force and Ca_i_^2+^ were increased at 1 Hz compared to 0.5 Hz beating rate (+10.0 ± 2.7% and +3.4 ± 0.8%, respectively), but started to diminish at 2 Hz (-14.2 ± 5.1% and -9.4 ± 2.3%, respectively). Further rate-dependent changes are depicted in Supplementary Table 2. As expected, systolic durations of force and Ca_i_^2+^ were shortened at higher beating rates, and diastolic relaxation and Ca_i_^2+^ sequestration were accelerated (tau of exponential decay). Interestingly, there was a very good correlation between the developed force and the Ca_i_^2+^ amplitude at the different beating rates and pre-beat intervals (Fig. 4 and Supplementary Fig. 1 and Supplementary Table 2).

**Fig. 5:**
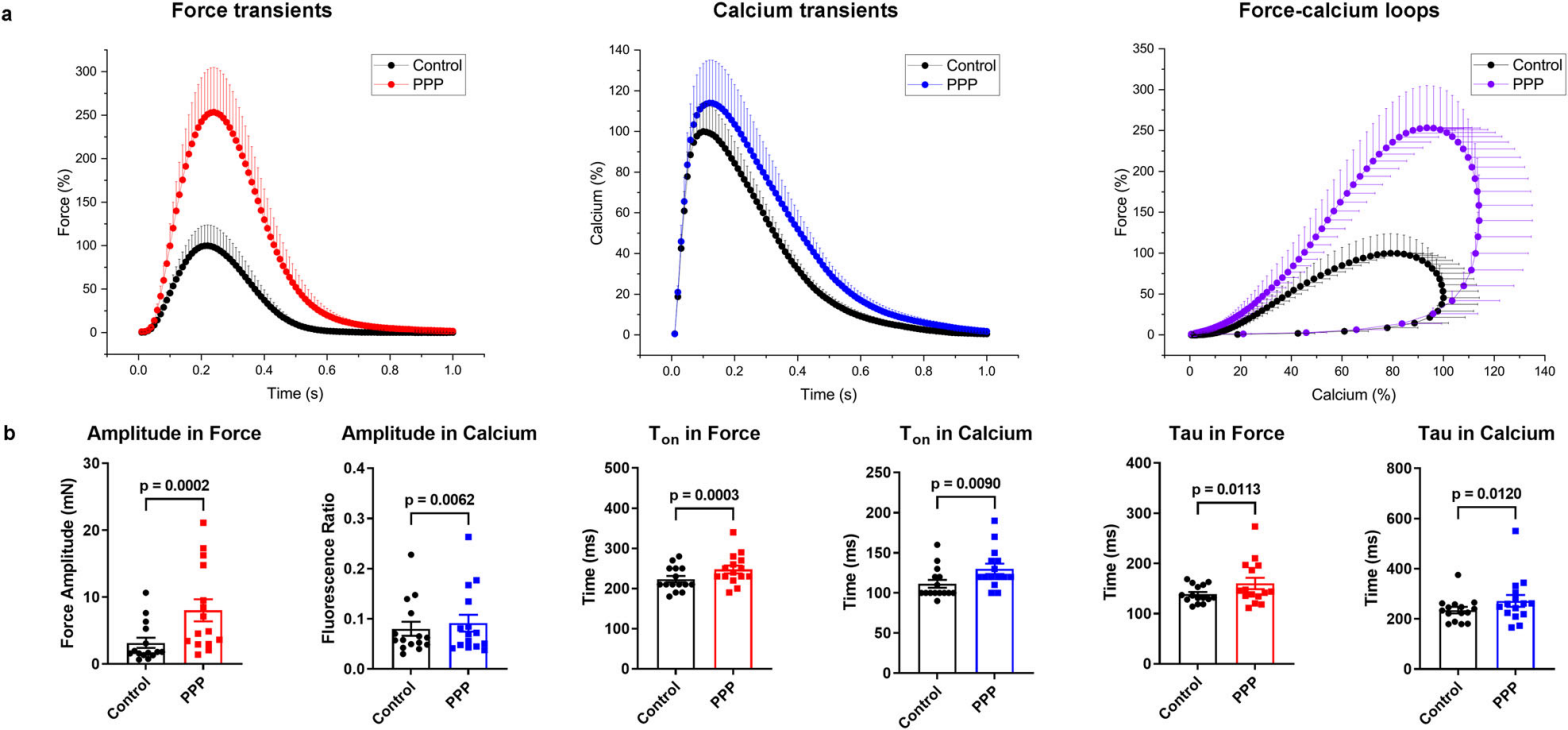
Alteration of force and calcium transients in post-pause potentiation. **a** Normalized average contraction force and calcium and force-calcium loops in human myocardial slices in post-pause potentiation (PPP, 12 s; *n* = 5 patients, 15 slices) at 0.5 Hz pacing frequency. **b** Amplitude (difference between peak and baseline values), T_on_, and tau in force and calcium transients in PPP (*n* = 5 patients, 15 slices). Data presented as mean and SEM. Statistical differences were calculated with paired Student *t* test with a significance cutoff of *p* < 0.05.

Diastolic rest is thought to increase contractility of the first subsequent beat by increasing the fraction of sarcoplasmic reticulum (SR) Ca^2+^ release^17^. After a 12-second rest, peak amplitude of force was much more enhanced (+153.6 ± 29.3%), than the amplitude of Ca_i_^2+^ (+14.0 ± 4.2%). Remarkably, the kinetics of relaxation and Ca_i_^2+^ decay were similarly retarded (tau +15.5 ± 5.1% and +16.0 ± 5.2%, respectively). The force-calcium loops demonstrated a stronger contractility after a prolonged resting period, which reflects increased SR Ca^2+^ release (Fig. 5, Supplementary Fig. 1 and Supplementary Table 2).

**Fig. 6:**
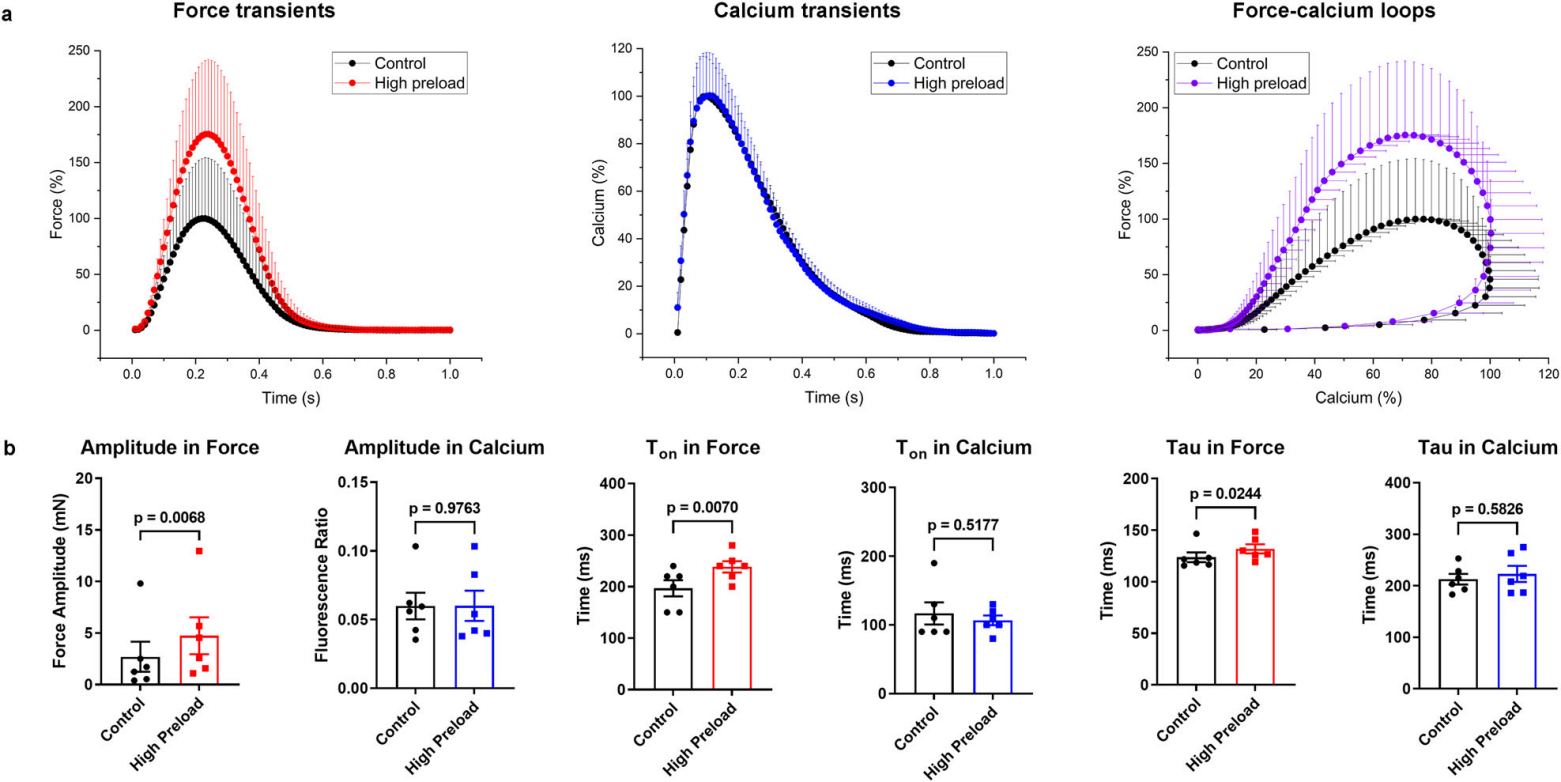
Alteration of force and calcium transients under high mechanical preload. **a** Normalized average contraction force and calcium and force-calcium loops in human myocardial slices under high preload (5000 µN, *n* = 3 patients, 6 slices). **b** Amplitude (difference between peak and baseline values), T_on_, and tau in force and calcium transients under high preload (*n* = 3 patients, 6 slices). Data presented as mean and SEM. Statistical differences were calculated with paired Student *t* test with a significance cutoff of *p* < 0.05.

High mechanical preload increased diastolic myocardial length and enhanced contractility, causing a significant increase in force amplitude (+75.5 ± 15.6%) but no change in peak Ca_i_^2+^. High preload significantly retarded relaxation (tau, +6.5 ± 1.9%), but did not affect Ca_i_^2+^ sequestration. The force-calcium loops demonstrated Ca_i_^2+^ was unaltered but force was dramatically raised in highly preloaded myocardium (Fig. 6, Supplementary Fig. 1 and Supplementary Table 2).

### Response of contractility and Ca_i_^2+^ to pharmacological interventions

**Fig. 7:**
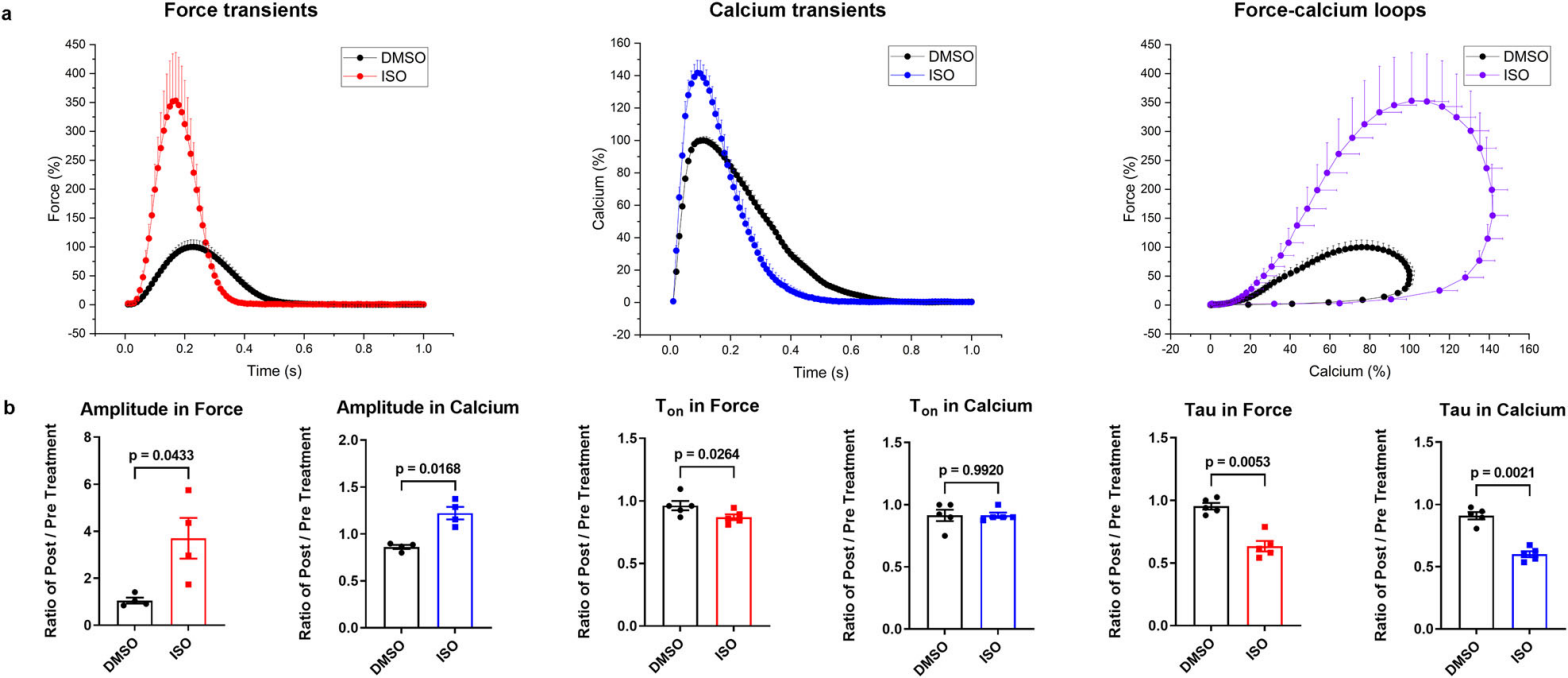
Response of force and calcium to isoprenaline (ISO). **a** Normalized average contraction force and calcium and force-calcium loops of human myocardial slices in the presence of DMSO (0.1%, *n* = 4 patients, 4 slices) or ISO (0.5 µM, *n* = 4 patients, 4 slices) at 0.5 Hz pacing frequency. **b** Relative changes of amplitude, T_on_, and tau in force and calcium transients of myocardial slices after treatment with DMSO or ISO (amplitude, *n* = 4 patients, 4 slices; T_on_ and tau, *n* = 5 patients, 5 slices in force and calcium, respectively). Data are depicted as means ± SEM. Statistical differences were calculated with paired Student *t-*tests. A *p* < 0.05 was defined as the significance cutoff.

The beta-selective adrenergic agonist isoprenaline has intense positive inotropic effects, which significantly increased amplitudes of force and Ca_i_^2+^ (+253.0 ± 64.9%, +41.7 ± 7.4%), and accelerated relaxation and Ca_i_^2+^ sequestration (tau, -33.4 ± 5.4% and -34.0 ± 4.3%, respectively). Diastolic force and Ca_i_^2+^ were not changed by isoprenaline. The force-calcium loops showed a more rapid increase in the early phase and an accelerated decay in the terminal phase, which demonstrated a faster contractile response to Ca_i_^2+^ and higher force and Ca_i_^2+^ amplitude in presence of isoprenaline (Fig. 7 and Supplementary Fig. 2 and Supplementary Table 3).

**Fig. 8:**
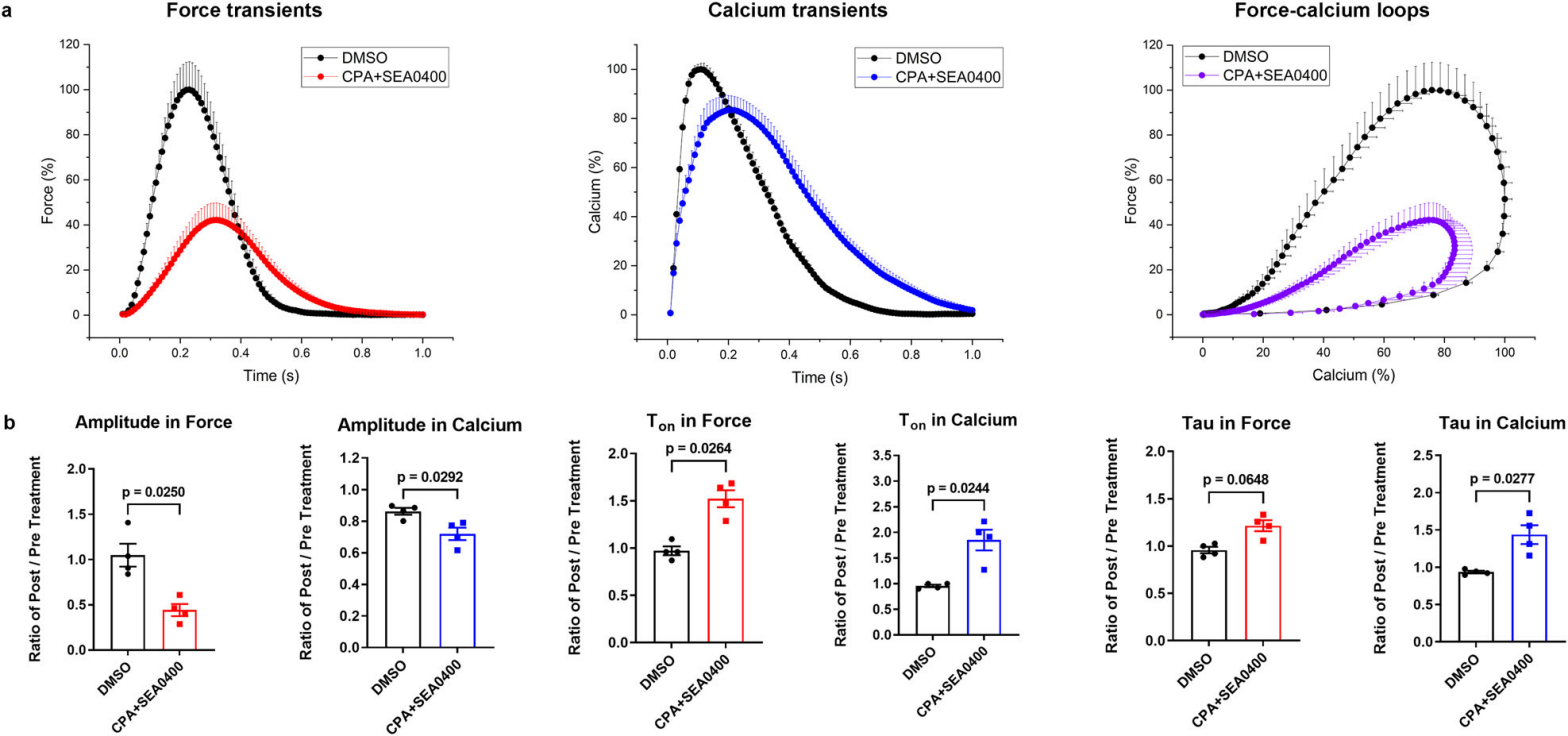
Response of force and calcium to CPA and SEA0400. **a** Normalized average contraction force and calcium and force-calcium loops of human myocardial slices in the presence of DMSO (0.1%, *n* = 4 patients, 4 slices), or CPA and SEA0400 (25 µM, 25 µM, *n* = 4 patients, 4 slices) at 0.5 Hz pacing frequency. **b** Relative changes of amplitude, T_on_, and tau in force and calcium transients of myocardial slices after treatment with DMSO, or CPA and SEA0400 (amplitude, *n* = 4 patients, 4 slices; T_on_ and tau, *n* = 5 patients, 5 slices in force and calcium, respectively). Data are depicted as means ± SEM. Statistical differences were calculated with paired Student *t-*test with a significance cutoff of *p* < 0.05.

**Fig. 9:**
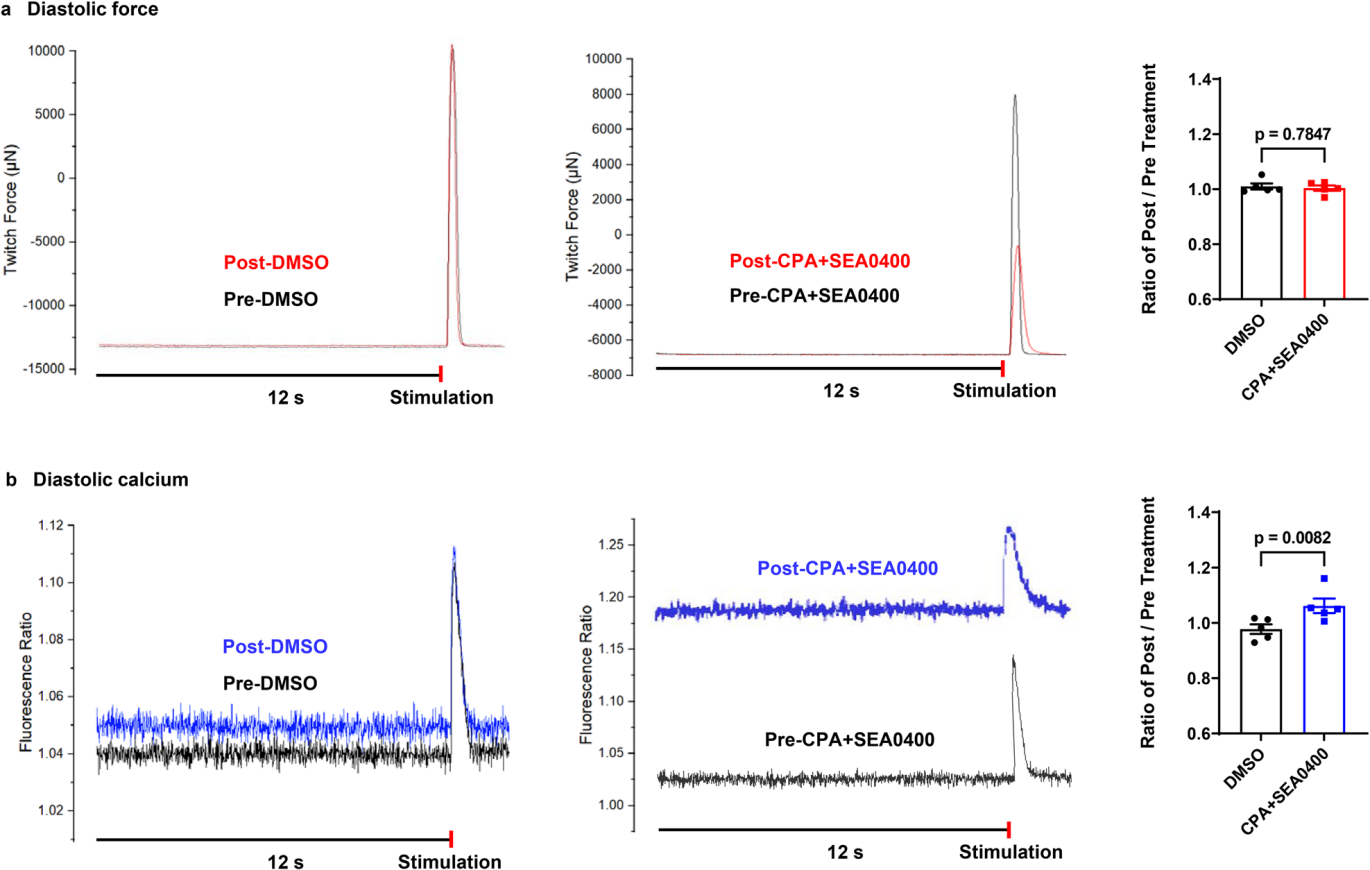
Diastolic force and calcium in presence of CPA and SEA0400 in human myocardial slices. **a**, **b** Representative images and statistics of diastolic force and calcium before and after DMSO (0.1%, *n* = 5 patients, 5 slices) or CPA + SEA0400 (25 µM, 25 µM, *n* = 5 patients, 5 slices) treatments. Data are presented as means ± SEM. Statistical analysis were performed with paired Student *t*-test with a significance cutoff of *p* < 0.05.

Combined inhibition of SERCA and NCX by cyclopiazonic acid (CPA) and SEA0400 was applied to decelerate the diastolic decay of Ca_i_^2+^. Consequently, greatly increased tau of relaxation and Ca_i_^2+^ decay (+25.7 ± 6.6% and +55.5 ± 9.5%, respectively) and increased diastolic Ca_i_^2+^ (+8.6 ± 1.6%) were observed. In addition, the treatment reduced contractility and peak Ca_i_^2+^ (-57.8 ± 12.0% and -16.6 ± 3.6%, respectively), potentially due to depletion of the sarcoplasmic Ca^2+^store. The force-calcium dependency describes the reduction of both in the presence of CPA and SEA0400, and resembles quite the opposite of the effects of isoprenaline (Fig. 8 and Fig. 9 and Supplementary Fig. 2 and Supplementary Table 3).

## Discussion

While freshly isolated myocardium is suitable for acute measurements, and for characterization of a preexisting disease^6–8^, long-term cultured human myocardial slices provide an opportunity to perform chronic interventions in vitro. For instance, signaling cascades of remodeling or processes of recovery and their effects on ECC in the setting of heart failure might be investigated. For comprehensive analysis of ECC, the simultaneous and synchronized assessment of force and Ca_i_^2+^ was the major goal of this study. This goal implies that uncoupling reagents which are frequently applied to suppress motion artifacts in optical mapping studies cannot be used^18,19^. Alternative methods to deal with motion artifacts rely on image-based motion correction^20^ or will reference the Ca^2+^-sensitive signal to a second one that is subjected to an identical movement (ratiometric approach). Our method is based on the latter, since this approach is able not only to cope with motion artifacts, but also to correct contraction-related shifts in the optical focus distance and inhomogeneities of dye loading (Supplementary Fig. 4). The use of CalRed eliminates the requirement of UV-light excitation that exists for the most common ratiometric Ca^2+^-sensitive dyes, such as Fura-2 and Indo-1^21^. We suppose that excitation of CalRed with visible light helps to reduce light toxicity and fast photobleaching. With regard to temporal stability of measurements, the CalRed dye offers the additional advantage of extensive persistence within the cells of interest, which in our case permitted repetitive measurements of Ca_i_^2+^ before and after drug treatment, i.e. at a 30 min interval. Alternative dyes may require the addition of transport inhibitors (e.g. probenecid) for prolongation of cellular retention^22^. In general, the Ca^2+^-sensitive dye CalRed has been found to provide advantages such as high signal-noise ratio (Supplementary Fig. 5a), post-exposure fluorescence recovery (Supplementary Fig. 5b, c), sensitivity to diastolic Ca_i_^2+^ (Fig. 9), and low toxicity, thus permitting continued cultivation of a tissue slice even after completion of an examination protocol.

Demonstrating the above advantages, the present study provides a method for the real-time and simultaneous detection of Ca_i_^2+^ and contractility in heart slices. The main findings are (1) simultaneous comparison of force and Ca_i_^2+^ kinetics and characterization of force-calcium relationship in human failing myocardium; (2) verification of force and Ca_i_^2+^ responses to high stimulation frequencies, post-rest, high preload, and specific compounds in heart failure, which are consistent with the known mechanisms; and (3) synchronized determination of force and Ca_i_^2+^ in human myocardial diastole, and (4) the possibility of intraindividual comparisons enabled by repeated measurements before and after treatments.

The force-calcium and ECC relationships were characterized by force and Ca_i_^2+^ kinetics (peak amplitude, tau, T_50on_, T_on_, T_50off_, T_off_, TD_50,_ and TD) as well as force-calcium loops in this study. The potential mechanism of ECC in myocardium has been identified in previous animal models^6,23^. In contraction phase I, Ca^2+^-induced activation of myofilaments and binding of cross-bridges cause a slight acceleration in contraction as Ca_i_^2+^ release is triggered and the Ca_i_^2+^ signal rapidly peaks. During contraction phase II, although the Ca_i_^2+^ concentration already decreases, the contraction continues to rise. In fact, the most significant fraction of the total force is generated during phase II. In the subsequent relaxation phase (phase III), the continued decrease in Ca_i_^2+^ reverses the contribution of the cross-bridge force, leaving only the passive tissue tension (preload) during myocardial diastole, which is in line with the results found here (Fig. 3).

Relevant alterations of ECC were verified by customized stimulation (force-frequency relation and post-pause potentiation) and high mechanical preload as physiological interventions in the approach we have developed. In previous studies, we could demonstrate a negative force-frequency relationship based on disturbed intracellular calcium handling^24,25^ and reduced t-tubule density^26^, as well as a low maximum capture rate^27^ as pathophysiological characteristics of failing myocardium. The present results show a maximum of contractility at 60 bpm beating rate and an appropriate shortening of kinetic parameters of Ca_i_^2+^ and force (tau, T_on_, T_50off_, T_off_, TD_50_, and TD) at higher heart rates. This may indicate that Ca^2+^ cycling has reverted to a more physiological state during the 3 weeks of cultivation prior to the experiments^25^. PPP represents the accumulation of Ca_i_^2+^ in the SR during electrical stimulation pauses and is strongly related to the capability of the SR to store and release calcium^17^. Thus, it depends on the relative contributions of SERCA and NCX to diastolic Ca_i_^2+^ removal from the cytosol^28–30^. Our results show that PPP induces a substantial enhancement in the amplitude of Ca_i_^2+^ and force and a prolongation of the time-dependent parameters (tau, Ton, T_50off_, and TD_50_) in both transients after a short rest (12 s). However, the sustained increase in post-rest twitch tension with increasing rest intervals is limited in failing human myocardium, which may be related to intracellular Ca^2+^ disorders and altered SR release in cardiomyocytes^17,31,32^. Furthermore, the approach we have developed also offers the potential to methodically investigate the effects of mechanical preload at different tensions on force and Ca_i_^2+^. High preload distinctly enhanced myocardial contractility but did not affect Ca_i_^2+^ appreciably, which might result from an increase in Ca^2+^ sensitivity to troponin C, a decrease in myofilaments spacing, and the formation of more actin-myosin cross-bridges, leading to more contractility^33–36^, whereas there is barely any effect on SR Ca^2+^ loading^37^.

The implementation of specific compounds serves as a potent validation of the applicability and feasibility of our developed methods, and the pharmacological response of ECC. Our results demonstrate that isoprenaline significantly enhanced the amplitude of myocardial Ca_i_^2+^ and contractility and also substantially shortened most time-dependent parameters of the calcium and force transients (tau, T_50off_, T_off_, TD_50,_ TD; T_on_ in force). However, isoprenaline had no remarkable impact on diastolic calcium and force. These results are in accordance with cAMP-dependent activation of protein kinase A, inducing an increase in SR calcium load and sequestration via increased L-type Ca^2+^ current (*I*_Ca_) influx, ryanodine receptor phosphorylation and disinhibition of SERCA^38,39^. Conversely, combined inhibition of SERCA and NCX (CPA + SEA0400) decreased Ca^2+^ removal from the cytosol as well as reduced SR Ca^2+^, reflected in a substantial increase in diastolic Ca_i_^2+^ and a significant reduction of the systolic amplitudes of Ca_i_^2+^ and force. Furthermore, it prolonged the associated temporal kinetics (tau, T_50on_, T_on_, T_50off_, T_off_, TD_50,_ and TD), which is essentially consistent with previous reports^40–43^.

More importantly, multiple subsequent observations of force and Ca_i_^2+^ are feasible to assess chronic effects of interventions using our method. This will allow to investigate long-term effects of certain interventions as well as effects that require long-term interventions. Possible applications include modifications of gene expression, chronic arrhythmic or tachycardic pacing, long-term application of pharmacological agents or chronic mechanical stimulation. Our approach can also be considered for the simultaneous force and Ca_i_^2+^ determination of myocardial slices in other species (e.g. pig, rat, and mice), engineered heart tissues, and possibly even in non-myocardial contractile tissues (e.g. vascular smooth muscle, tracheal smooth muscle, and skeletal muscle).

Our study reveals the following potential limitations for the use of CalRed: (1) In order to enhance the CalRed penetration into deeper myocardial layers and to acquire the sustained recoverable fluorescence for the multi-exposure, CalRed required an extended loading time to enhance the fluorescence intensity without affecting contractility, which increases the experimental complexity (Supplementary Fig. 5d-f); (2) Autofluorescence was not stable during measurements so that it cannot be predicted by detection prior to CalRed loading, and cannot be deducted independently in the presence of calcium fluorescence after CalRed loading and multiple exposures (Supplementary Fig. 6a); (3) Contraction force and Ca_i_^2+^ transients were measured as average values of the total slice and specific slice areas, respectively. Although the heterogeneity of the tissue is disregarded in this way, a reasonable correlation may exist between both parameters because tissue locations displaying clear Ca_i_^2+^ transients may be expected to predominantly contribute to overall contraction force (Supplementary Fig. 6b).

## Methods

### Human heart slice acquisition and long-term cultivation

Left-ventricular myocardial specimens of failing hearts that had been excised at transplantation were obtained from the Thoracic and Cardiovascular Surgery Clinic of the Heart and Diabetes Center in Bad Oeynhausen, Germany, and the Cardiac Surgery Clinic of the University Hospital in Munich, Germany, with the patients’ informed consent for the scientific use of the resected tissue. The collection and use of tissue have been approved by the ethics review committee of the Ruhr-University Bochum and the ethics commission of the Medical Faculty of the Ludwig-Maximilians-University Munich. The study was performed in accordance with the ethical standards outlined in the 1964 Declaration of Helsinki and the Data Protection Act and subsequent amendments, and all ethical regulations relevant to human research participants were followed. Upon excision, tissue samples were immediately (<15 min) transferred to cold (4 °C) cardioplegic buffer (136 mM NaCl, 5.4 mM KCl, 1 mM MgCl_2_, 0.33 mM NaH_2_PO_4_, 10 mM glucose, 0.9 mM CaCl_2_, 30 mM 2,3-butadione-2-monoxime, 5 mM HEPES, pH = 7.4), and were kept in this buffer at 4 °C during transportation (<36 h) and the slicing procedure^9^.

Myocardial slice preparation and BMCCs have been described in previous studies^9,44^. After precision cutting in 4 °C slicing buffer, myocardial slices were attached to plastic triangles, trimmed to a width of 7 mm, and mounted in BMCCs with integrated magnetic force sensor, stimulation electrodes, and linear drive for preload adjustment. The myocardial slices were cultivated with mechanical preload (1000–1200 µN), and continuous field stimulation (0.5 Hz) in M199 medium (#31150-022, Gibco), supplemented with ITS (Insulin-Transferrin-Selenium, 1%, #41400045, Gibco), Pen/Strep (Penicillin & Streptomycin solution, 1%, #P0781, Sigma-Aldrich), β-ME (β-mercaptoethanol, 50 µM, #A1108-0100, Applichem), and hydrocortisone (20 nM, #H0888, Sigma-Aldrich). All chambers were connected with the rocking platforms (60 rpm, 15° tilt angle) in a standard incubator (37 °C, 5% CO_2_, 20% O_2_, 80% humidity). Two-thirds of the culture medium was exchanged every 48 h. After 3–4 weeks of stabilization, myocardial slices were used for the simultaneous measurements of Ca_i_^2+^ fluorescence and contractility.

### CalRed loading and experimental design

To eliminate optical interference of phenol red, the medium was exchanged against a phenol red-free equivalent (#11043-023, Gibco, Thermo Fisher) 30 min prior to the experiment. 2.4 µL of 10 mM CalRed R525/650 AM (CalRed, dissolved in DMSO, #20590, AAT Bioquest, USA) and 12 µL of 10% Pluronic F-127 (dissolved in water, #P2443, Sigma-Aldrich, USA) were pre-mixed with 2385.6 µL phenol red-free medium in a thermo-mixer (#5382EO410758, Eppendorf, Germany) at 37 °C for 20 min, resulting in final concentrations of 10 µM and 0.05%, respectively. Myocardial slices were loaded with CalRed for 15 h to enhance the CalRed penetration into the intramyocardial layers and to acquire a sustained recoverable fluorescence for repeated exposures. Prior to the experiments, slices were equilibrated for 30 min in fresh medium to remove CalRed ester and to restore myocardial contractility.

Functional measurements were performed on a heated (37 °C) microscopic stage that permitted continuous force measurements and programmable stimulation of slices within their individual biomimetic chambers. Various stimulation modalities (beating rates, stop intervals) were produced with an automated 60 s stimulation protocol that was executed at baseline. Following 20 min equilibration in the incubator, isoprenaline (ISO, 0.5 µM, #I5627, Sigma-Aldrich, USA), SERCA inhibitor CPA (25 µM, #ab120300, Abcam), NCX inhibitor SEA0400 (25 µM, 2-[4-[(2,5-difluorophenyl) methoxy] phenoxy]-5-ethoxyaniline, #SML2054, Sigma-Aldrich), DMSO (0.1%, dimethyl sulfoxide, #276855, Sigma-Aldrich), or high mechanical preload (5000 µN) were applied respectively. All the specific compounds (ISO, CPA, and SEA0400) were dissolved in DMSO as stock solutions. After 10 min of further incubation, BMCCs were again placed on the microscopic stage, and all measurements were repeated. To continue the culture of the specimens, the compounds were washed out, and the mechanical preload was re-adjusted to normal (1000 µN) after the measurements.

### Synchronization of contractility recording and calcium imaging

Micro-Manager software (v1.4, MM Studio v1.4.23, MMCore v8.7.1, Device API v69, Module API v10, National Institutes of Health, USA)^45^ and MyoDish software (v2.0.7969.26226, InVitroSys GmbH, Germany) were used for the acquisition of force and Ca_i_^2+^ signals. The controller of the MyoDish cultivation system (model MD-01-01) was used to record the force signal of the BMCCs, and to generate impulses for electrical tissue stimulation and camera triggering. The various conditions of stimulation and imaging during the assessment of functional parameters were defined in a script that was executed by the MyoDish software (Supplementary Script). The script employed stimulation frequencies of 0.5-2 Hz and stimulation currents of 0-80 mA (exemplified in Fig. 2).

The preload-adjustable BMCCs with CalRed loaded myocardial slices were mounted on a custom-built imaging system based on an inverted microscope (Axiovert 35, 20x objective, Zeiss, Germany), an EM-CCD camera (Rolera EM-C^2^_,_ Q-imaging, USA), LED excitation (480 nm, Thorlabs, USA), and a wavelength-selective image splitter (Optosplit II, Cairn Research, UK) equipped with dual emission filters (525/30 nm and 670/50 nm) and a dichroic mirror (568 nm, all AHF GmbH, Germany). Force recording (400 Hz sampling rate) and programmed stimulation was equivalent to the functions of the biomimetic cultivation system (MyoDish, InVitroSys GmbH, Germany). Images were taken at 100 frames/s at a 251 × 250-pixel resolution (4 × 4 binning, 8 ms exposure time, 4000 EM-gain), and the camera was triggered in synchrony with the stimulation and force measurements by the MyoDish controller. For repeated measurements at identical slice areas, the biomimetic chambers were positioned on the microscopic stage by a custom-made motorized XY-drive. Locations of individual measurements were stored and retrieved using a stepper motor controller with corresponding software (bCNC, v0.9.14) (Fig. 1, Supplementary Fig. 3 and Supplementary Movie 1–3).

### Data analysis

Raw data of twitch force were converted to the Axon Binary File Format using the MyoDish Data File Converter (v1.1, InVitroSys GmbH, Germany), and were analyzed by either WinEDR (v3.9.7, University of Strathclyde, Scotland) or LabChart Reader (v8.1, AD Instruments, Australia). The fluorescence intensities at 525 nm and at 650 nm were obtained from the images representing either wavelength by calculating the mean pixel intensities of areas covering almost the full of the size of view (0.2 mm^2^) using ImageJ (v1.48, Java v1.6.0.31, National Institutes of Health, USA). The ratio of CalRed fluorescence at 525/650 nm was calculated without consideration of background fluorescence. This ratio is further addressed as an indicator of intracellular Ca^2+^ concentration, irrespective of its non-calibrated and non-linear feature. Twitch force and Ca_i_^2+^ at the same time point were synchronized and integrated, and all the parameters of Ca_i_^2+^ and force kinetics (peak amplitude, tau, T_50on_, T_on_, T_50off_, T_off_, TD_50,_ TD, diastolic force, diastolic Ca_i_^2+^) were analyzed by OriginPro (v2021, OriginLab Corporation, USA). Noise-reduction for raw data of force or Ca_i_^2+^ transient was processed by adjacent-averaging smoothing (moving mean filters). Fluorescence photobleaching was corrected by CalRed ratio at 650 / 525 nm emission. The time constant tau (*τ*) of Ca_i_^2+^ sequestration was calculated on the basis of single-exponential decay fitting, using the equation: p = p_0_ + A·exp (-τ/t), where p is percentage of the Ca_i_^2+^ or force transient, p_0_ is the curve baseline, *A* is amplitude of exponential, *t* is time. Signal-to-noise ratio (SNR) was defined as mean signal amplitude divided by the standard deviation of the baseline signal. Graphs depicting the time courses of force and Ca_i_^2+^ transients present each parameter with reference to its peak value under control conditions.

### Statistics and reproducibility

Statistical analysis was performed using GraphPad Prism (v7.04, GraphPad Software, USA). Statistical differences were calculated with paired or unpaired Student *t* test, and one-way ANOVA with Dunnett’s multiple comparisons posttest. Data are presented as mean ± SEM. A *p* < 0.05 was applied as significance cut-offs at all instances. All experiments were independently performed at least three times. More statistical details for each experiment are presented in the figure legends.

### Reporting summary

Further information on research design is available in the Nature Portfolio Reporting Summary linked to this article.

### Supplementary information

Supplementary Information

Description of Additional Supplementary Files

Supplementary Data

Supplementary Movie 1

Supplementary Movie 2

Supplementary Movie 3

Reporting Summary

## Data Availability

All data are available in the Supplementary Data or upon request to the corresponding author.

## Code availability

The command script defining the schedule of synchronized force measurements and Ca^2+^ imaging as executed by the MyoDish software (v2.0.7969.26226, InVitroSys GmbH, Germany) is provided as Supplementary Script in Supplementary Information.
