# Supplementary material for: Synchronous force and Ca2+ measurements for repeated characterization of excitation-contraction coupling in human myocardium
Source: Commun Biol. 2024 Feb 22;7:220. doi: 10.1038/s42003-024-05886-3 (PMC10884022; doi:10.1038/s42003-024-05886-3)
Supplement: Supplementary file 1 — Supplementary Information [file 42003_2024_5886_MOESM1_ESM.pdf]

SUPPLEMENTARY INFORMATION

Synchronous force and Ca^{2+} measurements for repeated characterization of excitation-contraction coupling in human myocardium

Zhengwu Sun[1], Kun Lu[2,3], Christine Kamla[2], Petra Kameritsch[1], Thomas Seidel[4], Andreas Dendorfer[1,3,*]

[1] Walter-Brendel-Centre of Experimental Medicine, University Hospital, Ludwig-Maximilians-University Munich, Munich, Germany.

[2] Department of Cardiac Surgery, University Hospital, Ludwig-Maximilians-University Munich, Munich, Germany.

[3] DZHK (German Center for Cardiovascular Research), Partner site Munich Heart Alliance, Munich, Germany.

[4] Institute of Cellular and Molecular Physiology, Friedrich-Alexander University Erlangen-Nürnberg, Erlangen, Germany.

* Correspondence: andreas.dendorfer@med.uni-muenchen.de

Table of contents

Supplementary Fig. 1……………………………………………………………………………3

Supplementary Fig. 2……………………………………………………………………………5

Supplementary Fig. 3…………………………………………………………………………....7

Supplementary Fig. 4……………………………………………………………………………9

Supplementary Fig. 5………………………………………………………… ……………….11

Supplementary Fig. 6…………………………………………………………………..………13

Supplementary Table 1………………………………………………………………………….15

Supplementary Table 2………………………………………………………………………….16

Supplementary Table 3………………………………………………………………………….17

Supplementary Script……………………………………………………………………..………18

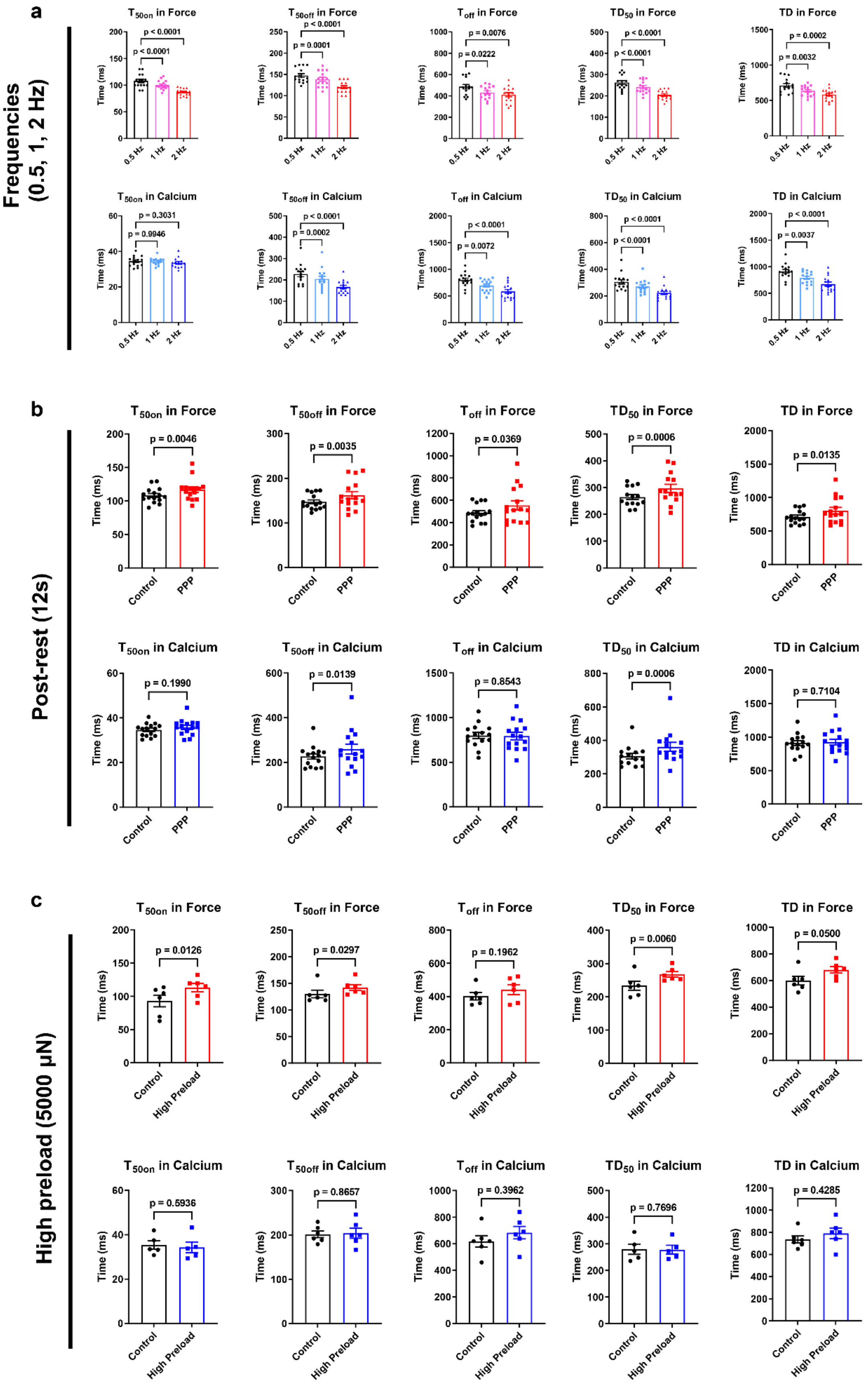
a
Frequencies (0.5, 1, 2 Hz)
T50on in Force
p < 0.0001
p < 0.0001
T50off in Force
p < 0.0001
p = 0.0001
Toff in Force
p = 0.0076
p = 0.0222
TD50 in Force
p < 0.0001
p < 0.0001
TD in Force
p = 0.0002
p = 0.0032
T50on in Calcium
p = 0.3031
p = 0.9946
T50off in Calcium
p < 0.0001
p = 0.0002
Toff in Calcium
p < 0.0001
p = 0.0072
TD50 in Calcium
p < 0.0001
p < 0.0001
TD in Calcium
p < 0.0001
p = 0.0037
Time (ms)
0.5 Hz
1 Hz
2 Hz
b
Post-rest (12s)
T50on in Force
p = 0.0046
T50off in Force
p = 0.0035
Toff in Force
p = 0.0369
TD50 in Force
p = 0.0006
TD in Force
p = 0.0135
T50on in Calcium
p = 0.1990
T50off in Calcium
p = 0.0139
Toff in Calcium
p = 0.8543
TD50 in Calcium
p = 0.0006
TD in Calcium
p = 0.7104
Time (ms)
Control
PPP
c
High preload (5000 μN)
T50on in Force
p = 0.0126
T50off in Force
p = 0.0297
Toff in Force
p = 0.1962
TD50 in Force
p = 0.0060
TD in Force
p = 0.0500
T50on in Calcium
p = 0.5936
T50off in Calcium
p = 0.8657
Toff in Calcium
p = 0.3962
TD50 in Calcium
p = 0.7696
TD in Calcium
p = 0.4285
Time (ms)
Control
High Preload

Supplementary Fig 1. Extended parameters of force and calcium analysis in human myocardium at various pacing modalities or high mechanical preload. a T_{50on}, T_{50off}, T_{off}, $TD_{50,}$ and TD at different pacing frequencies (0.5 Hz, 1 Hz, 2 Hz; n = 5 patients, 15 slices, except for T_{50on} in calcium, n = 5 patients, 14 slices). **b** T_{50on}, T_{50off}, T_{off}, $TD_{50,}$ and TD in post-pause potentiation (12s, n = 5 patients, 15 slices). **c** T_{50on}, T_{50off}, T_{off}, $TD_{50,}$ and TD under high preload (5000 μN, n = 3 patients, 6 slices). Data are depicted as means ± SEM. Statistical analysis was performed by one-way ANOVA with Dunnett's multiple comparisons test versus 0.5 Hz, or paired Student *t* test with a significance cutoff of $p < 0.05$.

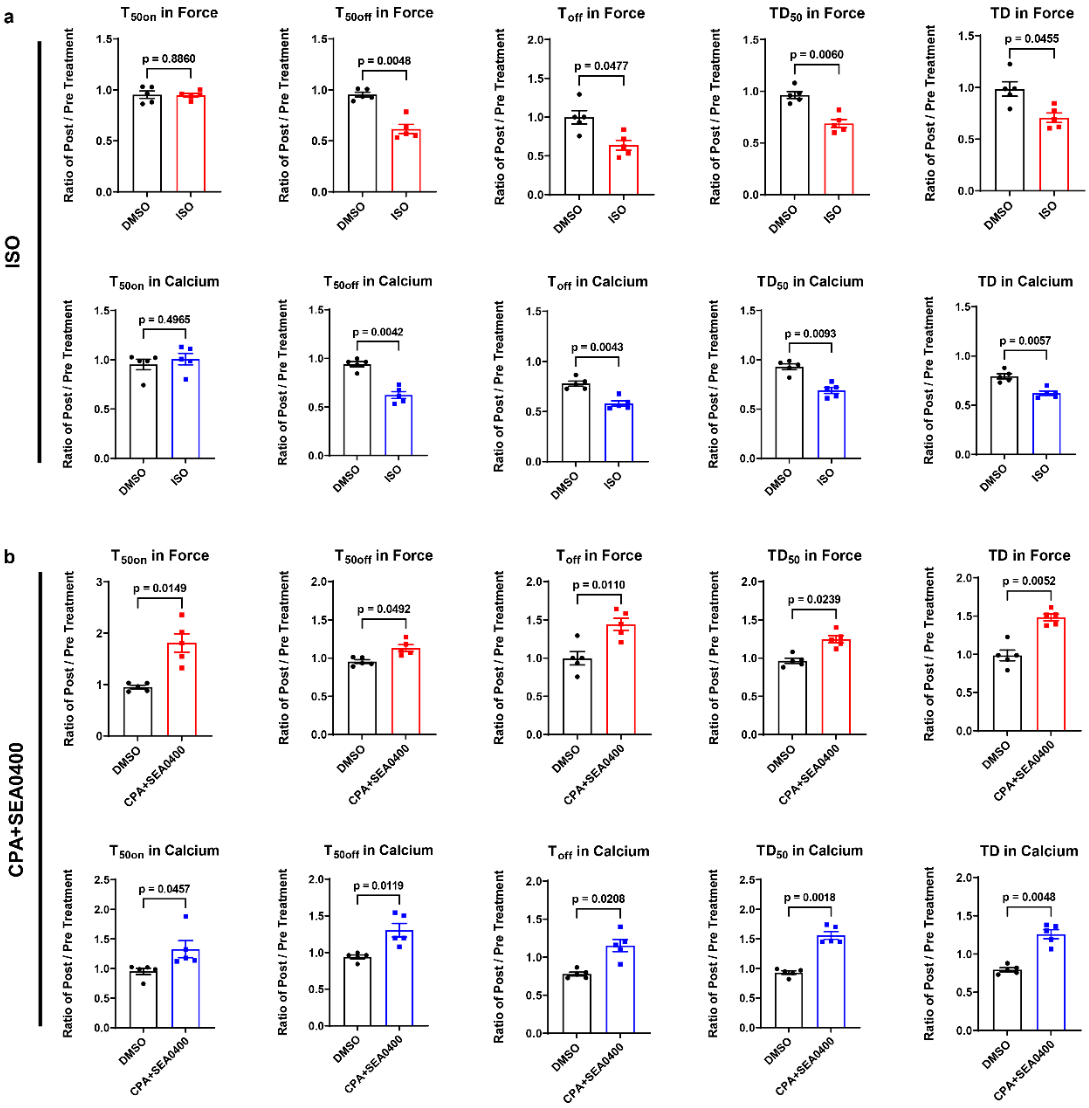
a
ISO
T50on in Force
p = 0.8860
T50off in Force
p = 0.0048
Toff in Force
p = 0.0477
TD50 in Force
p = 0.0060
TD in Force
p = 0.0455
T50on in Calcium
p = 0.4965
T50off in Calcium
p = 0.0042
Toff in Calcium
p = 0.0043
TD50 in Calcium
p = 0.0093
TD in Calcium
p = 0.0057
Ratio of Post / Pre Treatment
DMSO
ISO
b
CPA+SEA0400
T50on in Force
p = 0.0149
T50off in Force
p = 0.0492
Toff in Force
p = 0.0110
TD50 in Force
p = 0.0239
TD in Force
p = 0.0052
T50on in Calcium
p = 0.0457
T50off in Calcium
p = 0.0119
Toff in Calcium
p = 0.0208
TD50 in Calcium
p = 0.0018
TD in Calcium
p = 0.0048
Ratio of Post / Pre Treatment
DMSO
CPA+SEA0400

Supplementary Fig 2. Extended parameters of force and calcium analysis in human myocardium in response to isoprenaline (ISO) or CPA plus SEA0400. a Relative changes of T_{50on}, T_{50off}, T_{off}, TD_{50}, and TD in absence and presence of DMSO (0.1%, n = 5 patients, 5 slices) and ISO (0.5 µM, n = 5 patients, 5 slices). **b** Relative changes of T_{50on}, T_{50off}, T_{off}, TD_{50}, and TD in absence and presence of DMSO (0.1%, n = 5 patients, 5 slices) and CPA + SEA0400 (25 µM, 25 µM, n = 5 patients, 5 slices). Data are depicted as means ± SEM. Statistical analysis was performed by paired Student *t* test with a significance cutoff of $p < 0.05$.

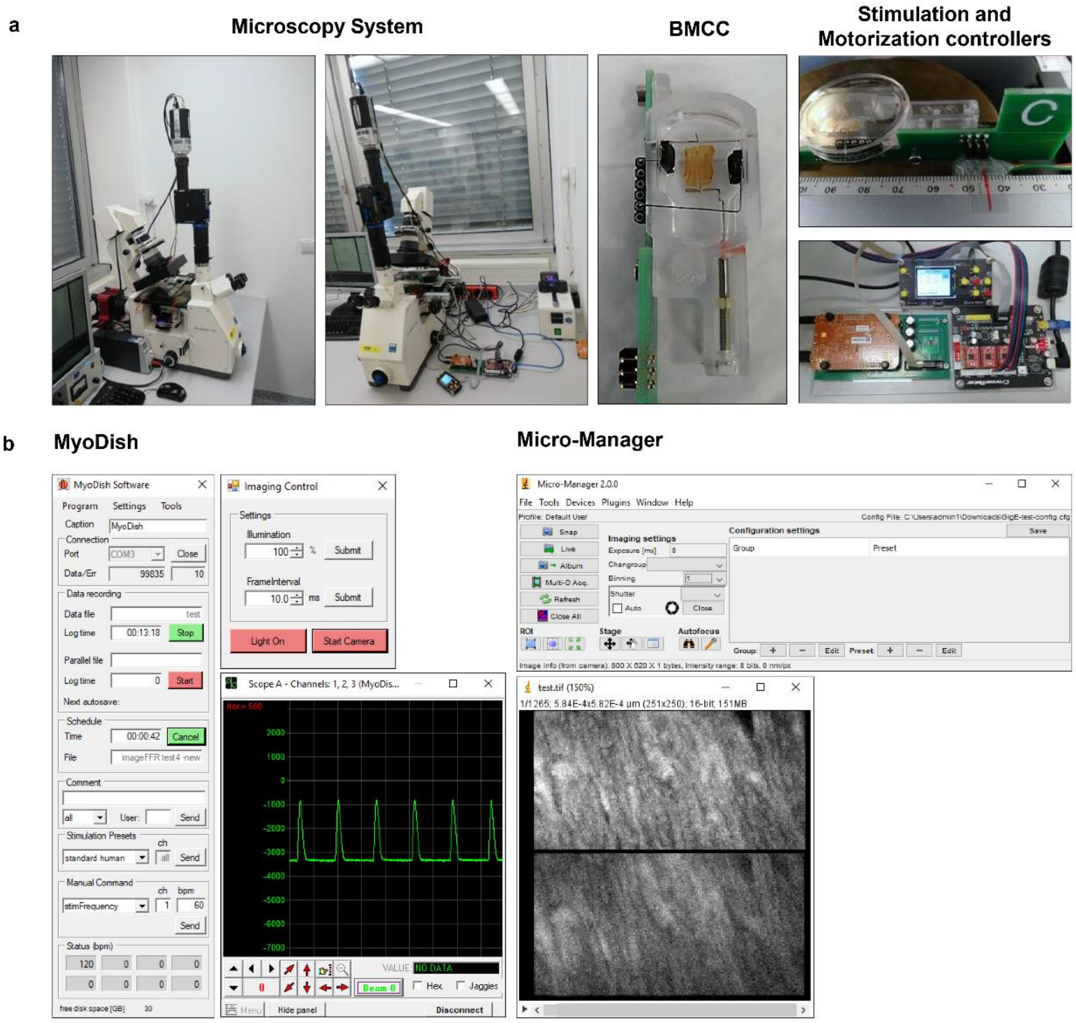
a
Microscopy System
BMCC
Stimulation and Motorization controllers
b
MyoDish
Micro-Manager

Supplementary Fig 3. Establishment of imaging system with stimulation for synchronized force and calcium measurements. a Components of imaging system: motorized fluorescent microscope with local computer, high-speed camera, 37°C thermostatic heating mat, and thermostatic light-proof cover; biomimetic culture chamber (BMCC); stimulation socket, stimulation controller and motorized stage controller. **b** Software required for synchronized force and calcium measurements, including MyoDish (twitch force recording and stimulation schedule) and Micro-Manager (calcium fluorescence imaging).

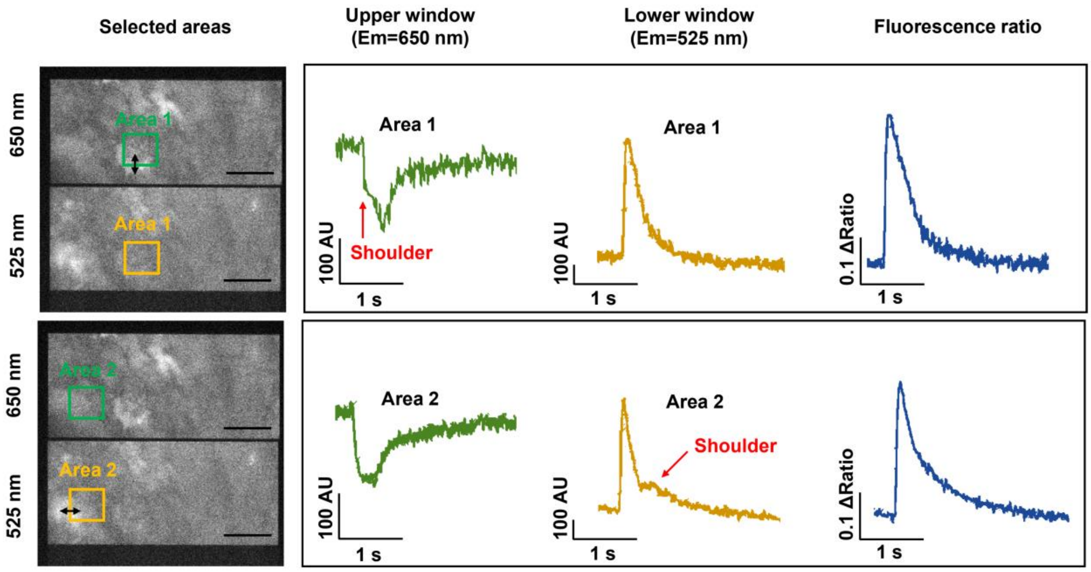

Selected areas
Upper window (Em=650 nm)
Lower window (Em=525 nm)
Fluorescence ratio
650 nm
525 nm
Area 1
Area 2
Shoulder
100 AU
1 s
0.1 ΔRatio

Supplementary Fig 4. Motion artifact correction using CalRed ratio in human myocardium. Representative images of motion artifacts at 650 nm and 525 nm emission (20× magnification, scale bar 135 μm), the movement of brightly fluorescent cluster of human myocardium during contraction induces shoulders with main calcium transients in selected areas (in and out of green area 1 and yellow area 2), which are corrected by calculation of CalRed fluorescence ratio. AU represents arbitrary units.

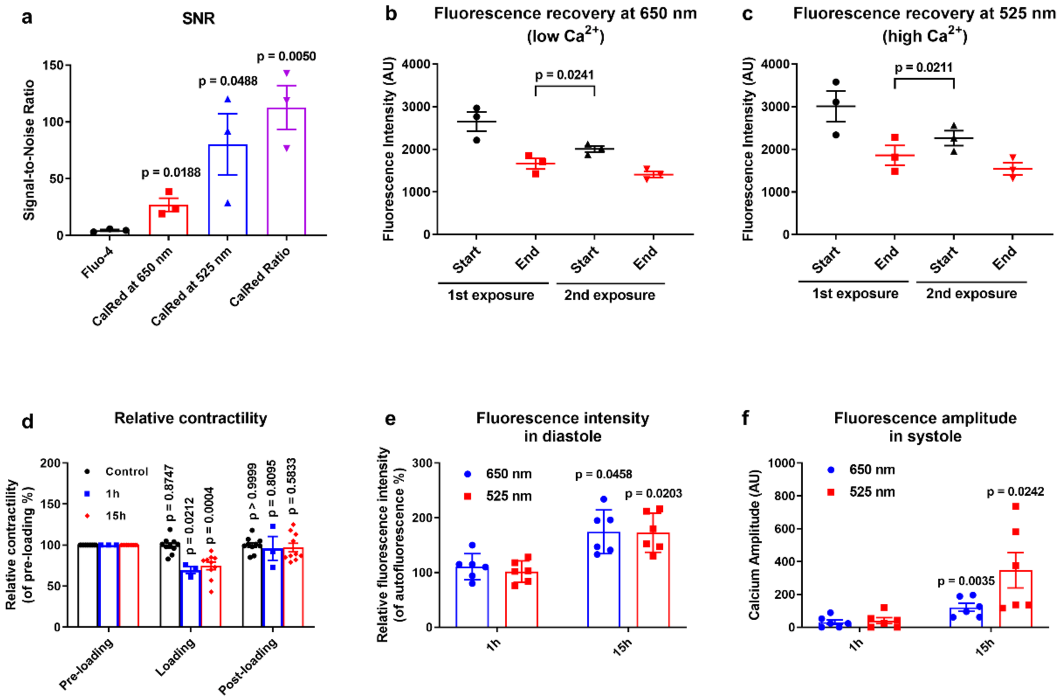
a
SNR
Signal-to-Noise Ratio
p = 0.0188
p = 0.0488
p = 0.0050
Fluo-4
CalRed at 650 nm
CalRed at 525 nm
CalRed Ratio
b
Fluorescence recovery at 650 nm
(low Ca^{2+})
Fluorescence Intensity (AU)
p = 0.0241
Start
End
1st exposure
2nd exposure
c
Fluorescence recovery at 525 nm
(high Ca^{2+})
p = 0.0211
d
Relative contractility
Relative contractility (of pre-loading %)
Control
1h
15h
p = 0.8747
p = 0.0212
p = 0.0004
p > 0.9999
p = 0.8095
p = 0.5833
Pre-loading
Loading
Post-loading
e
Fluorescence intensity
in diastole
Relative fluorescence intensity (of autofluorescence %)
650 nm
525 nm
p = 0.0458
p = 0.0203
1h
15h
f
Fluorescence amplitude
in systole
Calcium Amplitude (AU)
p = 0.0035
p = 0.0242

Supplementary Fig 5. CalRed loading kinetics and SNR and fluorescence recovery. a Signal-to-noise ratio (SNR) of CalRed compared to Fluo-4 (n = 4 patients, 8 slices for CalRed and Fluo-4). Fluorescence amplitude (peak – baseline). **b-c** Fluorescence recovery of CalRed at 650 nm and 525 nm**,** and respective fluorescent ratio (n = 3 patients, 3 slices) in two consecutive light exposures (1 min each, separated by 30 min) after CalRed loading for 15h**. d** Changes in twitch force during CalRed loading (n = 5 patients, 10 slices for control, n = 3 patients, 3 slices for 1h CalRed loading, n = 5 patients, 10 slices for 15 h CalRed loading). **e-f** Baseline fluorescence intensity in diastole and fluorescence amplitude in systole after CalRed loading for 1h or 15h (n = 6 patients, 6 slices). AU represents arbitrary units. Data are represented as means ± SEM. Statistical analysis was performed by paired *t* test, except for SNR where unpaired *t* test was used (a, Fluo-4 versus CalRed at 650 nm, Fluo-4 versus CalRed at 525 nm, Fluo-4 versus CalRed ratio; d, pre-loading versus loading, pre-loading versus post-loading; e, 1 h versus 15 h; f, 1 h versus 15 h). A $p < 0.05$ was defined as the significance cutoff.

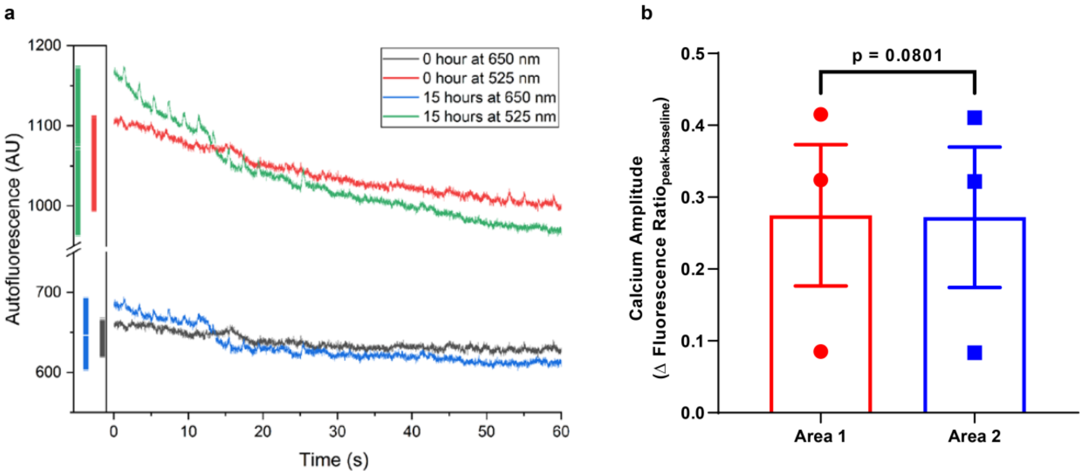
a
1200
1100
1000
700
600
Autofluorescence (AU)
0 hour at 650 nm
0 hour at 525 nm
15 hours at 650 nm
15 hours at 525 nm
0
10
20
30
40
50
60
Time (s)
b
p = 0.0801
0.5
0.4
0.3
0.2
0.1
0.0
Calcium Amplitude
(Δ Fluorescence Ratio$_{peak-baseline}$)
Area 1
Area 2

Supplementary Fig 6. Autofluorescence and calcium spatial heterogeneity. a Autofluorescence instability of unloaded myocardial slice with CalRed during the experimental stimulation protocol. Representative autofluorescence emission measured at 650 nm and 525 nm in myocardial slice during cultivation for 15h. The colored bars alongside vertical coordinates indicate fluorescence decay over the entire measurement period. AU represents arbitrary units. **b** Calcium amplitude (difference of CalRed fluorescence ratio between peak and baseline) of different areas (area 1 and area 2) from same slices (n = 3 patients, 3 slices). Statistical analysis was performed by paired *t* test with a significance cutoff of $p < 0.05$.

Supplementary Table 1. Patient characteristics

Age	Sex	Diagnosis	EF (%)
47	M	ICM	20%
60	M	RCM	50%
59	M	DCM	18%
39	M	ICM	5%
65	M	DCM	25%
37	M	DCM	15%
30	F	ACM	30%
53	M	ICM	10%
28	F	HCM	19%
51	F	DCM	20%

EF = Ejection fraction, M = Male, F = Female, ICM = Ischemic cardiomyopathy, RCM = Restrictive cardiomyopathy, DCM = Dilated cardiomyopathy. ACM = Arrhythmogenic cardiomyopathy, HCM = Hypertrophic cardiomyopathy.

Supplementary Table 2. Effects of programmed stimulation and high preload on force and calcium in human myocardium.

Kinetics	Category	High frequencies (1Hz)	High frequencies (2Hz)	Post-rest (12s)	High preload (5000 μN)
Amplitude	Force	+10.0±2.7%	-14.2±5.1%	+153.6±29.3%	+75.5±15.6%
	Calcium	+3.4±0.8%	-9.4±2.3%	+14.0±4.2%	+0.3±8.7%
Tau	Force	-5.6±0.4%	-16.8±1.5%	+15.5±5.1%	+6.5±1.9%
	Calcium	-9.2±1.6%	-24.1±1.9%	+16.0±5.2%	+4.9±7.6%
T_{50on}	Force	-7.2±1.2%	-19.4±2.2%	+8.0±2.3%	+21.5±5.6%
	Calcium	-3.6±3.1%	-6.0±3.2%	+3.0±2.4%	-3.1±4.8%
T_{on}	Force	-9.3±1.0%	-23.3±2.2%	+11.0±2.2%	+21.2±4.4%
	Calcium	-11.4±3.8%	-21.6±3.7%	+17.0±5.3%	-8.6±11.2%
T_{50off}	Force	-5.9±1.1%	-18.6±2.0%	+10.0±2.8%	+9.4±2.8%
	Calcium	-10.3±1.8%	-26.7±2.2%	+15.0±5.0%	+1.1±5.5%
T_{off}	Force	-11.2±3.8%	-15.5±4.4%	+14.0±5.8%	+10.0±6.1%
	Calcium	-13.4±3.8%	-27.1±3.3%	-1.0±3.9%	+10.5±10.3%
TD_{50}	Force	-8.2±0.8%	-22.2±1.9%	+12.1±2.4%	+14.5±2.9%
	Calcium	-11.4±1.4%	-27.1±1.4%	+18.2±3.7%	-0.5±1.6%
TD	Force	-10.6±2.6%	-17.9±3.2%	+13.0±4.4%	+13.6±4.8%
	Calcium	-13.2±3.3%	-26.4±2.9%	+1.0±3.5%	+7.5±7.9%

Listed values are average relative changes of force and calcium on peak amplitude, tau, T_{50on}, T_{on}, T_{50off}, T_{off}, TD_{50}, TD under high frequencies (amplitude in force and calcium, T_{50on} in calcium, n = 5 patients, 14 slices; tau, T_{on}, T_{50off}, T_{off}, TD_{50}, TD in force and calcium, T_{50on} in force, n = 5 patients, 15 slices, respectively), post-rest (n = 5 patients, 15 slices), and high preload (n = 3 patients, 6 slices). Green highlight marks statistically significant difference compared to 0.5 Hz or control. Data are presented as mean ± SEM. Statistical analysis was performed by one-way ANOVA with Dunnett's multiple comparisons test versus 0.5 Hz, or paired Student *t* test with a significance cutoff of $p < 0.05$.

Supplementary Table 3. Effects of pharmacological interventions on force and calcium in human myocardium.

**Kinetics**	**Category**	**Isoprenaline (0.5 µM)**	**CPA+SEA0400 (25 µM+25 µM)**
Amplitude	Force	+253.0±64.9%	-57.8±12.0%
	Calcium	+41.7±7.4%	-16.6±3.6%
Tau	Force	-33.4±5.4%	+25.7±6.6%
	Calcium	-34.0±4.3%	+55.5±9.5%
T_{50on}	Force	-0.6±3.7%	+90.1±19.7%
	Calcium	+5.8±6.9%	+39.4±12.3%
T_{on}	Force	-9.7±2.5%	+66.1±12.5%
	Calcium	0±3.5%	+120.9±26.2%
T_{50off}	Force	-35.4±5.6%	+18.7±6.0%
	Calcium	-33.8±5.2%	+38.8±7.9%
T_{off}	Force	-36.1±11.5%	+44.7±8.9%
	Calcium	-25.4±3.9%	+48.2±11.6%
TD_{50}	Force	-28.3±4.8%	+29.7±7.4%
	Calcium	-25.8±4.9%	+67.8±8.3%
TD	Force	-28.3±8.8%	+50.5±8.1%
	Calcium	-21.6±3.6%	+58.6±9.2%
Diastole	Force	-0.5±1.1%	-0.6±2.0%
	Calcium	+2.0±1.7%	+8.6±1.6%

Listed values are average relative changes of force and calcium on peak amplitude, tau, T_{50on}, T_{on}, T_{50off}, T_{off}, TD_{50}, TD, diastolic force and calcium in presence of isoprenaline or CPA+SEA0400 compared to DMSO (amplitude, n = 4 patients, 4 slices; tau, T_{50on}, T_{on}, T_{50off}, T_{off}, TD_{50}, TD, diastolic force and calcium n = 5 patients, 5 slices in force and calcium, respectively). Green highlight marks statistically significant difference compared to DMSO. Data are presented as mean ± SEM. Statistical analysis was performed by paired *t* test with a significance cutoff of $p < 0.05$.

Supplementary Script. MyoDish software (v2.0.7969.26226, InVitroSys GmbH, Germany)-based script to define the schedule of synchronized force measurements and Ca^{2+} imaging.

↓ Time of command execution in seconds

0; cameraState; 1; // switches on trigger impulses for the camera at 100 frames/s

1; stimPeriod; 2000; // defines 2000 ms repetition interval for delivery of stimulation pulse sequences

1; stimTime; 1; 0; // defines stimulation of channel 1 at the beginning of each sequence (time 0 ms)

11; stimPeriod; 2000; // defines stimulation sequence without stimulation event -> stop of stimulation

13; stimPeriod; 1000; // defines stimulation interval of 1000 ms -> 60 bpm beating rate

13; stimTime; 1; 0; // defines stimulation of channel 1 at the beginning of the sequence (time 0 ms)

25; stimPeriod; 1000; // defines stimulation sequence without stimulation event -> stop of stimulation

27; stimPeriod; 500; // defines stimulation interval of 500 ms -> 120 bpm beating rate

27; stimTime; 1; 0; // defines stimulation of channel 1 at the beginning of the sequence (time 0 ms)

37; stimCurrent; 1; 0; // defines 0 mA stimulation current for channel 1 -> stop of stimulation

39; stimPeriod; 2000; // presets the conditions for 30 bpm stimulation

39; stimTime; 1; 0; // defines stimulation of channel 1 at the beginning of the sequence (time 0 ms)

49; stimCurrent; 1; 80; // reestablishes stimulation by setting stimulation current to 80 mA

60; cameraState; 0; // stops imaging
