# Supplementary material for: Synchronous force and Ca2+ measurements for repeated characterization of excitation-contraction coupling in human myocardium
Source: Commun Biol. 2024 Feb 22;7:220. doi: 10.1038/s42003-024-05886-3 (PMC10884022; doi:10.1038/s42003-024-05886-3)
Supplement: Supplementary file 2 — Description of Additional Supplementary Files [file 42003_2024_5886_MOESM2_ESM.pdf]

Description of Additional Supplementary Files

File name: Supplementary Data
Description: The source data behind the graphs in the paper.

File name: Supplementary Movie 1
Description: Contraction of living myocardial slice with programmed stimulation in bright field (20× magnification, scale bar 135 μm) using Micro-manager software.

File name: Supplementary Movie 2
Description: Calcium signal of living myocardial slice with programmed stimulation (20× magnification, scale bar 135 μm) using Micro-manager software.

File name: Supplementary Movie 3
Description: Force recording of living myocardial slice with programmed stimulation using MyoDish software (X-axis is stimulation frequency, 0.5 Hz; Y-axis is contraction force, μN).
