# Supplementary material for: Synchronous force and Ca2+ measurements for repeated characterization of excitation-contraction coupling in human myocardium
Source: Commun Biol. 2024 Feb 22;7:220. doi: 10.1038/s42003-024-05886-3 (PMC10884022; doi:10.1038/s42003-024-05886-3)
Supplement: Supplementary file 7 — Reporting Summary [file 42003_2024_5886_MOESM7_ESM.pdf]

nature portfolio

Corresponding author(s): Andreas Dendorfer

Last updated by author(s): Jan 12, 2024

Reporting Summary

Nature Portfolio wishes to improve the reproducibility of the work that we publish. This form provides structure for consistency and transparency in reporting. For further information on Nature Portfolio policies, see our Editorial Policies and the Editorial Policy Checklist.

Statistics

For all statistical analyses, confirm that the following items are present in the figure legend, table legend, main text, or Methods section.

n/a	Confirmed	
☐	☒	The exact sample size (*n*) for each experimental group/condition, given as a discrete number and unit of measurement
☐	☒	A statement on whether measurements were taken from distinct samples or whether the same sample was measured repeatedly
☐	☒	The statistical test(s) used AND whether they are one- or two-sided<br>*Only common tests should be described solely by name; describe more complex techniques in the Methods section.*
☒	☐	A description of all covariates tested
☐	☒	A description of any assumptions or corrections, such as tests of normality and adjustment for multiple comparisons
☐	☒	A full description of the statistical parameters including central tendency (e.g. means) or other basic estimates (e.g. regression coefficient) AND variation (e.g. standard deviation) or associated estimates of uncertainty (e.g. confidence intervals)
☐	☒	For null hypothesis testing, the test statistic (e.g. *F*, *t*, *r*) with confidence intervals, effect sizes, degrees of freedom and *P* value noted<br>*Give P values as exact values whenever suitable.*
☒	☐	For Bayesian analysis, information on the choice of priors and Markov chain Monte Carlo settings
☒	☐	For hierarchical and complex designs, identification of the appropriate level for tests and full reporting of outcomes
☒	☐	Estimates of effect sizes (e.g. Cohen's *d*, Pearson's *r*), indicating how they were calculated

Our web collection on statistics for biologists contains articles on many of the points above.

Software and code

Policy information about availability of computer code

Data collection	Micro-Manager software (v1.4, MM Studio v1.4.23, MMCore v8.7.1, Device API v69, Module API v10, National Institutes of Health, USA), MyoDish software (v2.0.7969.26226, InVitroSys GmbH, Germany)
Data analysis	MyoDish Data File Converter (v1.1, InVitroSys GmbH, Germany), WinEDR (v3.9.7, University of Strathclyde, Scotland), LabChart Reader (v8.1, AD Instruments, Australia), ImageJ 278 (v1.48, Java v1.6.0.31, National Institutes of Health, USA), OriginPro (v2021, OriginLab Corporation, USA)

For manuscripts utilizing custom algorithms or software that are central to the research but not yet described in published literature, software must be made available to editors and reviewers. We strongly encourage code deposition in a community repository (e.g. GitHub). See the Nature Portfolio guidelines for submitting code & software for further information.

Data

Policy information about availability of data

All manuscripts must include a data availability statement. This statement should provide the following information, where applicable:

- Accession codes, unique identifiers, or web links for publicly available datasets
- A description of any restrictions on data availability
- For clinical datasets or third party data, please ensure that the statement adheres to our policy

All data will be available upon request to the corresponding author.

Research involving human participants, their data, or biological material

Policy information about studies with human participants or human data. See also policy information about sex, gender (identity/presentation), and sexual orientation and race, ethnicity and racism.

Reporting on sex and gender	7 patients were of male sex, and 3 patients were of female sex. Gender is unknown.
Reporting on race, ethnicity, or other socially relevant groupings	Not relevant due to low number of participants.
Population characteristics	Not relevant due to low number of participants.
Recruitment	Tissue was aquired with agreement from any patient undergoing heart transplantation.
Ethics oversight	Full identity is given in the manuscript.

Note that full information on the approval of the study protocol must also be provided in the manuscript.

Field-specific reporting

Please select the one below that is the best fit for your research. If you are not sure, read the appropriate sections before making your selection.

☒ Life sciences ☐ Behavioural & social sciences ☐ Ecological, evolutionary & environmental sciences

For a reference copy of the document with all sections, see nature.com/documents/nr-reporting-summary-flat.pdf

Life sciences study design

All studies must disclose on these points even when the disclosure is negative.

Sample size	Sample size were defined based on various observations from previous experiments.
Data exclusions	No data was excluded from analysis, except in one case in which quantitative analysis was impeded by pulsus alternans.
Replication	For all the experiments, no less than 3 experiments were performed.
Randomization	Samples were allocated to experimental groups without any previous selection.
Blinding	Experimental data with hidden sample code was analyzed by investigators.

Reporting for specific materials, systems and methods

We require information from authors about some types of materials, experimental systems and methods used in many studies. Here, indicate whether each material, system or method listed is relevant to your study. If you are not sure if a list item applies to your research, read the appropriate section before selecting a response.

Materials & experimental systems

n/a	Involved in the study	
☒	☐	Antibodies
☒	☐	Eukaryotic cell lines
☒	☐	Palaeontology and archaeology
☒	☐	Animals and other organisms
☐	☒	Clinical data
☒	☐	Dual use research of concern
☒	☐	Plants

Methods

n/a	Involved in the study	
☒	☐	ChIP-seq
☒	☐	Flow cytometry
☒	☐	MRI-based neuroimaging

Clinical data

Policy information about clinical studies

All manuscripts should comply with the ICMJE guidelines for publication of clinical research and a completed CONSORT checklist must be included with all submissions.

Clinical trial registration	Does not apply.
Study protocol	Does not apply.
Data collection	Does not apply.
Outcomes	Does not apply.
